# Sialic acid blockade inhibits the metastatic spread of prostate cancer to bone

**DOI:** 10.1016/j.ebiom.2024.105163

**Published:** 2024-05-20

**Authors:** Kirsty Hodgson, Margarita Orozco-Moreno, Emily Archer Goode, Matthew Fisher, Rebecca Garnham, Richard Beatson, Helen Turner, Karen Livermore, Yuhan Zhou, Laura Wilson, Eline A. Visser, Johan FA. Pijnenborg, Nienke Eerden, Sam J. Moons, Emiel Rossing, Gerald Hysenaj, Rashi Krishna, Ziqian Peng, Kyla Putri Nangkana, Edward N. Schmidt, Adam Duxfield, Ella P. Dennis, Rakesh Heer, Michelle A. Lawson, Matthew Macauley, David J. Elliott, Christian Büll, Emma Scott, Thomas J. Boltje, Richard R. Drake, Ning Wang, Jennifer Munkley

**Affiliations:** a Newcastle University Centre for Cancer, Newcastle University Institute of Biosciences, Newcastle upon Tyne NE1 3BZ, UK; b The Mellanby Centre for Musculoskeletal Research, Division of Clinical Medicine, The University of Sheffield, Sheffield, UK; c Centre for Inflammation and Tissue Repair, UCL Respiratory, Division of Medicine, University College London (UCL), Rayne 9 Building, London WC1E 6JF, UK; d Cellular Pathology, The Royal Victoria Infirmary, Queen Victoria Road, Newcastle upon Tyne NE1 4LP, UK; e Newcastle University Centre for Cancer, Translational and Clinical Research Institute, Newcastle University, Paul O’Gorman Building, Newcastle upon Tyne NE2 4HH, UK; f Synthetic Organic Chemistry, Institute for Molecules and Materials, Radboud University, Nijmegen, the Netherlands; g GlycoTherapeutics B.V., Nijmegen, the Netherlands; h Synvenio B.V., Nijmegen, the Netherlands; i Department of Chemistry, University of Alberta, Edmonton, AB T6G 2G2, Canada; j Department of Medical Microbiology and Immunology, University of Alberta, Edmonton, AB T6G 2E1, Canada; k International Centre for Life, Biosciences Institute, Newcastle University, Newcastle Upon Tyne NE1 3BZ, UK; l Department of Urology, Freeman Hospital, The Newcastle upon Tyne Hospitals NHS Foundation Trust, Newcastle upon Tyne NE7 7DN, UK; m Biomolecular Chemistry, Institute for Molecules and Materials, Radboud University Nijmegen, the Netherlands; n Department of Cell and Molecular Pharmacology, Medical University of South Carolina, Charleston, SC, USA; o Leicester Cancer Research Centre, Department of Genetics and Genome Biology, University of Leicester, LE2 7LX, UK

**Keywords:** Prostate cancer, Bone metastasis, Glycans, Sialylation, Sialic acid, Therapeutics

## Abstract

**Background:**

Bone metastasis is a common consequence of advanced prostate cancer. Bisphosphonates can be used to manage symptoms, but there are currently no curative treatments available. Altered tumour cell glycosylation is a hallmark of cancer and is an important driver of a malignant phenotype. In prostate cancer, the sialyltransferase ST6GAL1 is upregulated, and studies show ST6GAL1-mediated aberrant sialylation of *N*-glycans promotes prostate tumour growth and disease progression.

**Methods:**

Here, we monitor ST6GAL1 in tumour and serum samples from men with aggressive prostate cancer and using *in vitro* and *in vivo* models we investigate the role of ST6GAL1 in prostate cancer bone metastasis.

**Findings:**

ST6GAL1 is upregulated in patients with prostate cancer with tumours that have spread to the bone and can promote prostate cancer bone metastasis *in vivo*. The mechanisms involved are multi-faceted and involve modification of the pre-metastatic niche towards bone resorption to promote the vicious cycle, promoting the development of M2 like macrophages, and the regulation of immunosuppressive sialoglycans. Furthermore, using syngeneic mouse models, we show that inhibiting sialylation can block the spread of prostate tumours to bone.

**Interpretation:**

Our study identifies an important role for ST6GAL1 and α2-6 sialylated *N*-glycans in prostate cancer bone metastasis, provides proof-of-concept data to show that inhibiting sialylation can suppress the spread of prostate tumours to bone, and highlights sialic acid blockade as an exciting new strategy to develop new therapies for patients with advanced prostate cancer.

**Funding:**

Prostate Cancer Research and the 10.13039/100014599Mark Foundation For Cancer Research, the 10.13039/501100000265Medical Research Council and 10.13039/501100000771Prostate Cancer UK.

**Research in context**

**Evidence before this study**

Bone metastasis is a common, debilitating and incurable consequence of advanced prostate cancer. New treatments for bone metastasis are urgently needed and could significantly impact patient quality of life and survival rates. Aberrant glycosylation is a hallmark of cancer and new treatments targeting tumour-associated glycans are currently being tested in several clinical trials. A common change in prostate tumour cell glycosylation is an increase in larger branched α2-6 sialylated *N*-glycans, driven by the sialyltransferase ST6GAL1. ST6GAL1 is overexpressed in several cancer types and plays a fundamental role in tumour transformation, growth, metastasis, and immune evasion. In prostate cancer, ST6GAL1-mediated aberrant sialylation promotes tumour growth and is linked to disease progression, but the role of ST6GAL1 and sialylated *N*-glycans in prostate cancer metastasis has not yet been investigated.

**Added value of this study**

Here, we analyse tumour and serum samples from men with prostate cancer and show ST6GAL1 is upregulated in patients with aggressive disease, and specifically in men with bone metastasis. Using *in vivo* models, we find ST6GAL1 promotes the spread of prostate cancer to bone, and reveal the mechanisms involved are multi-faceted, and involve modification of the pre-metastatic nice towards bone resorption to promote the ‘vicious cycle’ of bone metastasis, as well as the regulation of M2 like macrophages and immunosuppressive sialoglycans. Furthermore, using syngeneic mouse models, we show that inhibiting sialylation can block the spread of prostate tumours to bone.

**Implications of all the available evidence**

There is an urgent need to develop new therapies for advanced prostate cancer and in particular treatments for patients with bone metastasis. This study provides proof-of-concept data to show that sialic acid blockade can inhibit the spread of prostate tumours to bone and highlights the targeting of aberrant sialylation in combination with existing treatments for prostate cancer as an exciting new strategy to develop urgently needed new therapies for advanced disease.

## Introduction

Prostate cancer is the second most common type of cancer among men and is a leading cause of cancer related deaths worldwide.^1^ While most patients with prostate cancer will receive a curative therapy, around a third of men progress to advanced metastatic disease.^2^ Among prostate cancer metastases, bone is the most common site of colonisation with up to 80% of patients with advanced prostate cancer diagnosed with tumours that have spread to bone.^3^ The 5-year survival rate for patients with prostate cancer bone metastasis is only 32%, whereas for patients with localised disease this is increased to 97%.^3^ Although treatments such as bisphosphonates are available to manage symptoms, it is not possible to cure prostate cancer bone metastasis, and the disease causes significant debilitating co-morbidities, including severe bone pain and fractures.^4^ In prostate cancer metastasis, malignant cells detach from the primary tumour, invade the blood or lymphatic system, and migrate to host tissues such as bone. Bone provides a matrix rich in factors to stimulate the growth of tumours and promote a vicious cycle of metastasis and bone pathology.^3^ The underlying mechanisms behind the high propensity of prostate cancer to metastasise to bone are poorly understood,^3^ and moving forward, an increased appreciation of the properties that facilitate the formation of the pre-metastatic bone niche and the colonisation and growth of prostate tumours in bone will pave the way for the development of urgently needed and lifesaving treatments for advanced prostate cancer.

Tumour cells often contain glycans with different structures and levels of expression compared to normal cells.^5^ This aberrant glycosylation is a hallmark of cancer^6^ and new therapies targeting tumour-associated glycans are currently being tested in several clinical trials.^7^^,^^8^ The most widely occurring changes in glycosylation linked to cancer progression include an enhancement of *N*-glycan branching due to increased activity of the glycosyltransferase MGAT5,^9^ and alterations to α2-6 sialylated *N*-glycans - a modification driven by the sialyltransferase enzyme ST6GAL1.^5^^,^^10^^,^^11^ ST6GAL1 is overexpressed in numerous types of cancer, including pancreatic, breast, ovarian and prostate cancer, and is associated with aggressive disease and poor patient prognosis.^10^^,^^12^^,^^13^ Upregulation of ST6GAL1 impacts oncogenic behaviours and plays a critical role in tumour transformation, survival, growth, metastasis, immune evasion and chemoresistance.^14^, ^15^, ^16^, ^17^ In prostate cancer, a rewiring of the tumour glycome has been linked to disease progression^18^^,^^19^ and both ST6GAL1 and larger branched sialylated *N*-glycans are increased in prostate tumour tissue relative to normal prostate tissue.^18^^,^^20^, ^21^, ^22^ We recently showed that ST6GAL1 levels are increased in the blood of men with prostate cancer and that upregulation of ST6GAL1 can promote prostate cancer cell invasion and tumour growth.^22^ However, although ST6GAL1 has been linked to more aggressive prostate tumours, to our knowledge the *in vivo* role of ST6GAL1 and sialylated *N*-glycans in prostate cancer metastasis remains a critical knowledge gap.

Here, using patient tissue samples, cell culture studies, and animal cancer models we show that ST6GAL1 is upregulated in men with prostate cancer bone metastasis, and reveal aberrant sialylation can promote the spread of prostate cancer to bone. Furthermore, we find the role of ST6GAL1 in aggressive prostate cancer likely involves modification of the bone pre-metastatic niche towards bone resorption to promote the vicious cycle, the promotion of M2 like macrophages and the regulation of immunosuppressive sialoglycans. Finally, using syngeneic mouse models, we show that sialic acid blockade using the newly developed C-5 carbamate sialyltransferase inhibitor P-SiaFNEtoc,^23^^,^^24^ significantly inhibits prostate cancer bone metastasis. Our findings identify ST6GAL1 and sialoglycans as major contributors to prostate cancer bone metastasis and highlight a new opportunity to combine sialic acid blockade with existing anti-androgen and immunotherapy treatments to develop urgently needed new combination therapies for advanced prostate cancer.

## Methods

### RT-qPCR

RNA extraction, cDNA synthesis and real-time PCR were as described previously.^25^, ^26^, ^27^

### Immunohistochemistry

Immunohistochemistry to detect ST6GAL1 protein in FFPE tissue sections was as described previously.^22^ Antigen retrieval was performed by pressure cooking the TMA for 90 s in 10 mM citrate pH 6.0 followed by staining the tissues with anti-ST6GAL1 antibody (Abgent, AP19891c) at a 1:6000 dilution. Nuclei were counterstained with haematoxylin. The TMA was scored by Helen Turner using the 0–300 Histoscore score method.^28^ Only epithelial cells were scored. Sections were scored based on their staining intensity with 0 being assigned to cells with absent staining, 1 to weak staining, 2 to moderate staining and 3 to strong staining. Within each staining intensity the percentage of epithelial cells (0–100%) with this staining intensity was assigned. This resulted in a Histoscore calculated from the following equation H = 0× (% of cells scored at 0) + 1× (% of cells scored at 1) + 2× (% of cells scored at 2) + 3× (% of cells scored at 3).

### ELISA assays

ST6GAL1 pre-validated sandwich ELISA assays were carried out as described previously.^22^ The cohort of 300 serum samples was kindly provided by Dr Colm Morrissey (University of Washington) via PCBN. Samples were prepared and stored using standard protocols. CSF1 ELISAs (Sigma, RAB0098) and IGFBP5 ELISAs (Raybiotech, ELH-IGFBP5) were carried out on conditioned media samples (prepared from cell lines as described previously^22^) as per the manufacturer’s instructions.

### Clinical cohorts

#### TMA cohort 1

A previously published 96 case TMA comprising 0.5 mm cores of prostate adenocarcinoma of different Gleason grades^26^ was purchased from US Biomax (PR1921b) (Supplementary Fig. S1c).

#### TMA cohort 2

**Fig. 1:**
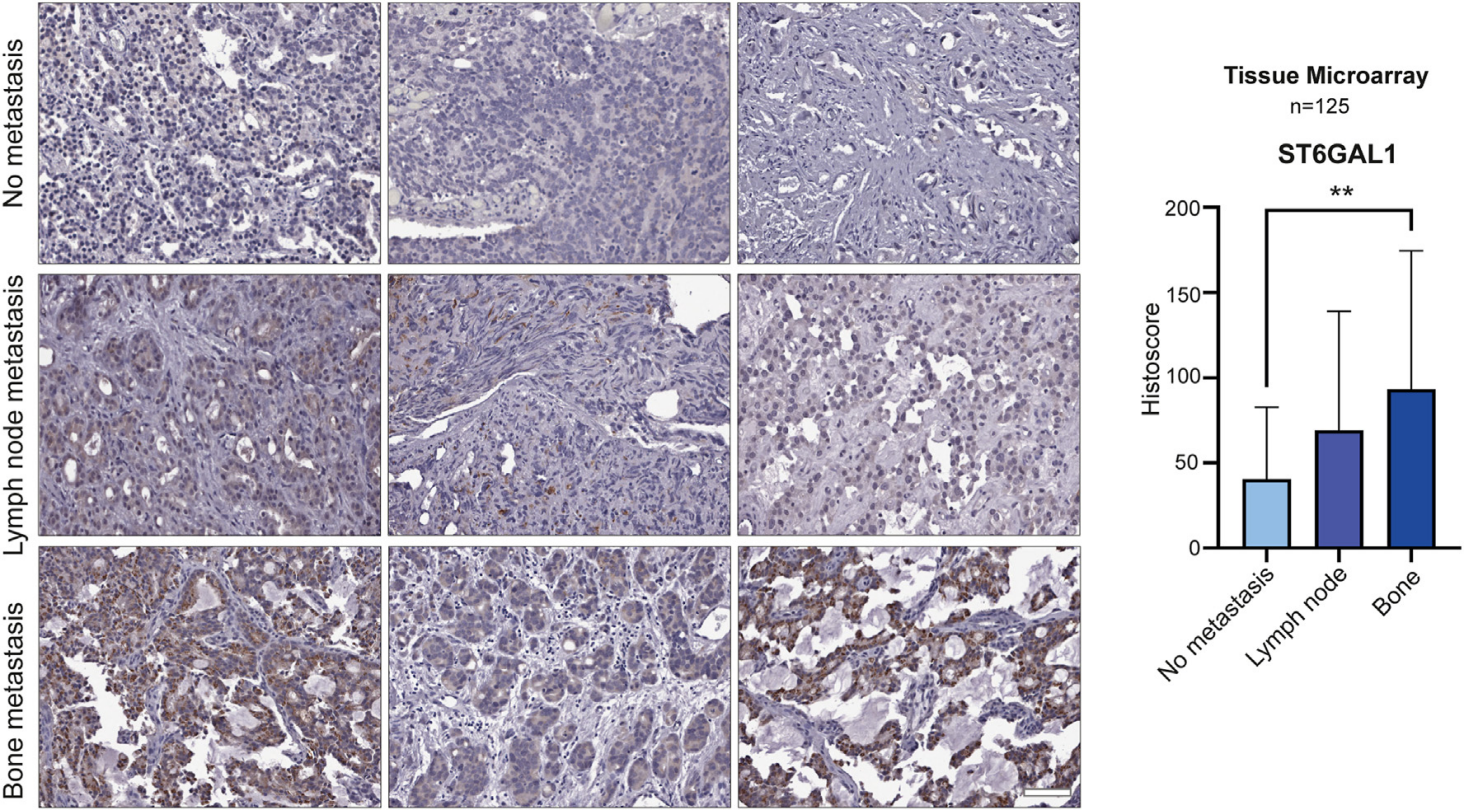
The sialyltransferase enzyme ST6GAL1 is upregulated in prostate cancer that has spread to the bone. (**a**) Immunohistochemistry analysis of a previously published 125 case tissue microarray (TMA)^29^ to compare ST6GAL1 levels in localised prostate cancer tumours and prostate cancer tissues presenting with metastasis to bone or lymph node (all biopsy samples were taken from the primary site). Further details for this cohort are provided in Supplementary Table S1. ST6GAL1 protein levels are 1.7-fold upregulated in patients with prostate cancer lymph node metastasis (unpaired t test, p = 0.1657) and 2.3-fold upregulated in patients with prostate cancer bone metastasis (unpaired t test, p = 0.0091). Scale bar is 100 μm.

An intermediate density 125 case TMA which has been previously published (Fig. 1a).^29^ The samples are from channel transurethral resections of a cohort of advanced prostate cancer cases, comprising of patients with either localised prostate cancer tumours or prostate tumours presenting with metastasis to bone or lymph node (all biopsy samples were taken from the primary site). More information on this cohort is provided in Supplementary Table S1.

#### Bone metastasis tissue samples from rapid autopsy

**Fig. 2:**
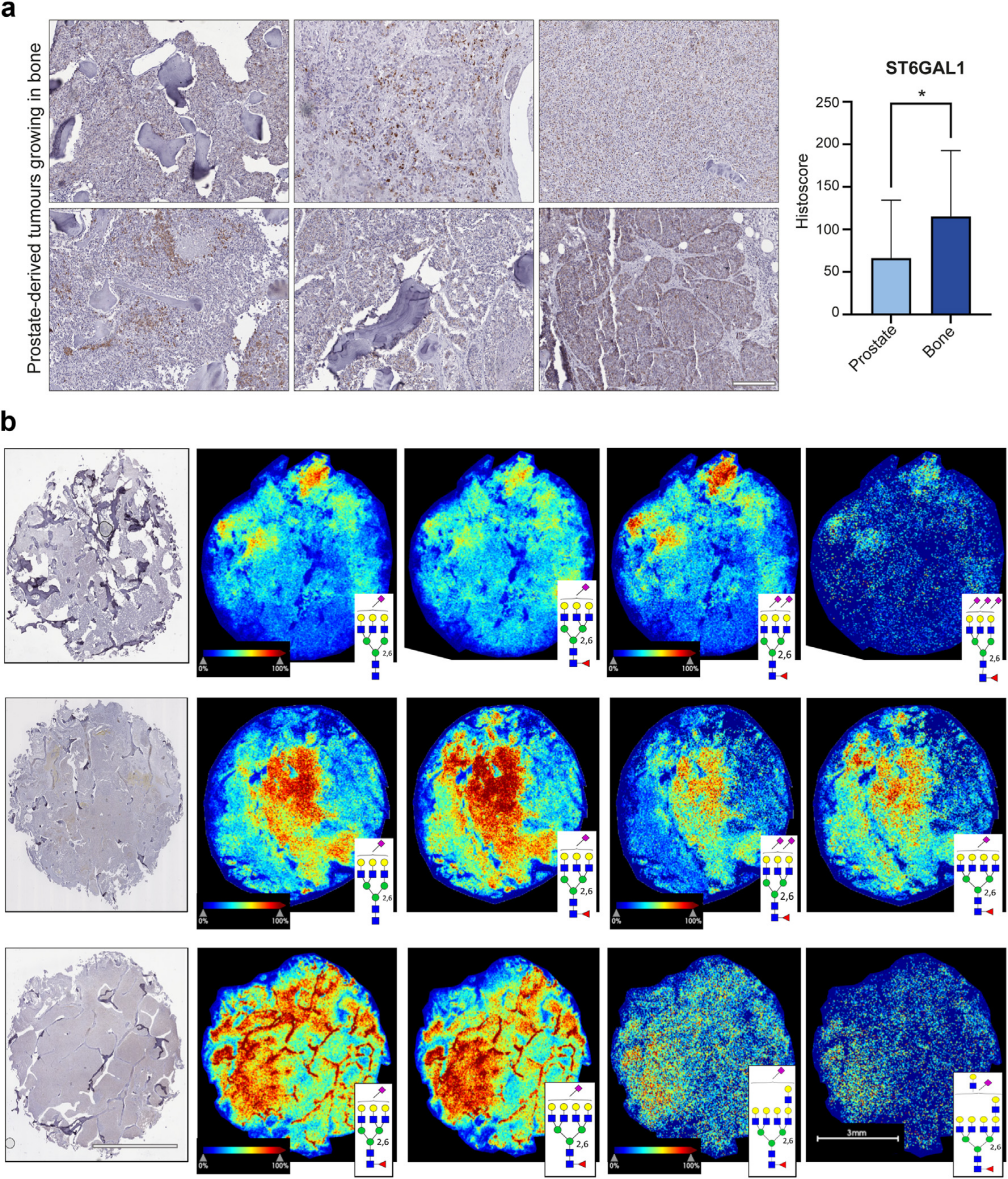
ST6GAL1 is upregulated in prostate-derived tumours growing in bone. (**a**) Immunohistochemistry analysis of ST6GAL1 levels in 20 rapid autopsy samples from prostate-derived tumours growing in bone compared to 53 localised prostate cancer tissue samples biopsied at the primary site (taken from the TMA analysed in Fig. 1). ST6GAL1 levels were 1.74-fold higher in prostate-derived tumours growing in bone compared to tumours from the primary site (unpaired t test, p = 0.0103). Scale bar is 200 μm. (**b**) *N*-glycan MALDI imaging mass spectrometry analysis of all 20 bone metastases tissue samples. Three bone tumours with high immunostaining levels of ST6GAL1 are shown in the first image of row, with representative α2-6 sialylated *N*-glycan images that co-localize to ST6GAL1-stained regions. Scale bar for IHC and MALDI images is 3 mm.

20 cases of rapid autopsy FFPE tissue samples from prostate-derived tumours growing in bone were provided by Dr Colm Morrissey (University of Washington) via the Prostate Cancer Biorepository Network (PCBN) (Fig. 2). Biopsies of metastatic bone sites were obtained from patients with CRPC within hours of death using a cordless drilling trephine (DeWalt Industrial Tool) and model 2422-51-000 trephine (DePuy). Bone cores were fixed in 10% neutral buffered formalin, decalcified with 10% formic acid and paraffin embedded.

#### Serum samples

**Fig. 3:**
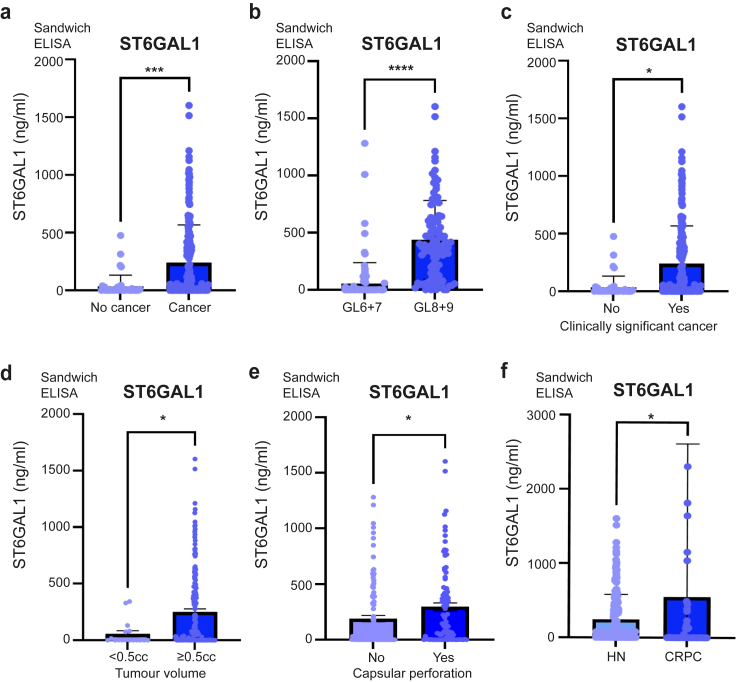
ST6GAL1 is upregulated in the blood of men with aggressive prostate cancer. Analysis of ST6GAL1 protein levels in a cohort of 300 serum samples using pre-validated sandwich ELISA assays, including 40 men given a ‘no cancer’ diagnosis, 100 men with low grade prostate cancer (Gleason grade 6–7), 100 men with high grade prostate cancer (Gleason grade 8–9), and 60 men with metastatic CRPC. More information on this cohort is provided in Supplementary Table S1. (**a**) ST6GAL1 serum levels were 7.3-fold higher in men diagnosed with prostate cancer relative to men without prostate cancer (n = 240, unpaired t test, p < 0.0001). (**b**) Levels of serum ST6GAL1 levels were 6.7-fold higher in men with high grade prostate cancer compared to men with low grade disease (n = 200, unpaired t test, p < 0.0001). (**c**) Serum ST6GAL1 levels were 10.6-fold higher in men with clinically significant prostate cancer compared to non-clinically significant prostate cancer defined according to the PI-RADS v2.1 guidelines^30^ (n = 200, unpaired t test, p = 0.143). (**d**) ST6GAL1 serum levels were 4.4-fold higher in men with a prostate tumour volume ≥0.5 cc compared to those with a tumour volume <0.5 cc (where a tumour volume of ≥0.5 cc is defined as clinically significant)^31^^,^^32^ (n = 200 unpaired t test, p = 0.0174). (**e**) The level of serum ST6GAL1 was 1.55-fold higher in prostate tumours with capsular perforation compared to tumours not involving or extending beyond the prostate gland (n = 200, unpaired t test, p = 0.0394). (**f**) Levels of serum ST6GAL1 were 2.24-fold higher in men with metastatic CRPC (58/60 of whom had metastasis to bone) compared to men with hormone naïve (HN) disease (n = 260, unpaired t test, p = 0.046).

300 serum samples were kindly provided for this project by Dr Colm Morrissey (University of Washington) via PCBN (Fig. 3). All samples were taken from patients who had undergone prostatectomies except the no cancer patients. All of the Gleason scores were from the time of radical prostatectomy. The ‘no cancer’ diagnosis patients were screened for prostate cancer at the University of Washington and found to be low risk. More information on this cohort is provided in Supplementary Table S1. Samples were prepared and stored via standard protocols.

### *N*-glycan MALDI-IMS and sialic acid stabilization

The 20 rapid autopsy bone metastasis prostate tissue slides were incubated with dimethylamine in dimethyl sulfoxide as previously described to amidate α2-6 sialylated *N*-glycans, followed by a second amidation reaction with propargylamine for stabilizing α2-3 sialylated *N*-glycans.^33^ Antigen retrieval, PNGase F digestion and analysis on a timsTOF fleX trapped ion mobility separated QTOF mass spectrometer (Bruker Corp., Bremen, Germany) was performed using a standardised protocol.^22^^,^^34^ Spectra and tissue images were annotated in SCiLS Lab software by matching peaks against an in-house *N*-glycan database that included amidated sialic acid isomers.

### Cell culture and creation of stable and transient cell lines

LNCaP (RRID:CVCL_0395), CWR22RV1 (RRID:CVCL_1045), PC3 (RRID:CVCL_0035), DU145 (RRID:CVCL_0105), RM1 (RRID:CVCL_B459) and TRAMPC2 cells (ATCC: CRL-2731) were cultured as described previously.^25^^,^^35^^,^^36^ The stable cell lines used in the study were created using lentiviral transduction. For knockdown of ST6GAL1, previously validated stable cell lines were used.^22^ For overexpression of ST6GAL1, Lentifect Purified lentiviral particles were purchased from Tebu-Bio (ST6GAL1 217LPP-M0351-Lv242-050-S and negative control 217LPP-NEG-Lv242-025-C). Transductions were carried out according to the manufacturer’s instructions using an MOI of 5. For the metastasis study, PC3 cells were transduced with Firefly Luciferase Lentivirus (BPS Bioscience, 79692-H) at a MOI of 1 in media containing 5 μg/ml polybrene. Stable cell lines were selected with 200 μg/ml hygromycin, and then further selected for ST6GAL1 overexpression using the methods described above. siRNA KD of *St6gal1* in mouse cell lines was performed by seeding 3000 cells per well in 8 well chamber slides. After 24, 48 and 72 h, cells were transfected with MISSION esiRNA targeting st6gal1 (Eupheria Biotech, EMU069151) or negative control RLUC (Renilla Luciferase) (Eupheria Biotech, EHURLUC) following the Lipofectamine RNAiMAX manufacturer’s protocol (Invitrogen, 13778). Briefly, esiRNA (100 ng) was diluted in 25 μl Opti-MEM I reduced serum medium (Invitrogen, 3198062) and mixed with 0.5 ul Lipofectamine RNAiMAX diluted in 25 μl Opti-MEM. The si-RNA lipid complex was added to the cells after 20 min room temperature incubation in a final volume of 251 μl. Cells were fixed for Siglec ligand profiling at 96 h. Luciferase expression was measured with the Luciferase Assay System (Promega, E1500). All of the cell lines used were validated using STR profiling and tested monthly for mycoplasma contamination.

### Sialyltransferase inhibitors

P-SiaFNEtoc was synthesised as described previously^23^ (compound 10).

### Mouse models

#### PC3 bone metastasis study

Six-week old male BALB/cAnNCrl immunocompromised (athymic nude) mice (RRID:MGI:2683685) were purchased from Charles River (Kent, UK) and housed in a controlled environment in Optimice cages (Animal Care Systems, Colorado, USA) randomly located in the cage rack with a 12 h light/dark cycle at 22 °C with ad libitum water and 2018 Teklad Global 18% protein rodent diet containing 1.01% Calcium (Harlan Laboratories, UK). Twenty mice were randomised into two groups to receive single-cell suspensions of 1 × 10^5^ PC3 (control or ST6GAL1 overexpressing) cells/100 μL PBS via injection into the left cardiac ventricle of mice (intracardiac injection). Tumour progression was monitored weekly based on bioluminescence using the *in vivo* imaging systems (IVIS, PerkinElmer, Cambridge, UK) for 6 weeks and the images were blinded for data analysis.

#### TRAMPC2 tumour xenografts

TRAMPC2 cells^36^ were pre-treated with 64 μM P-SiaFNEtoc or vehicle (1.875% DMSO) *in vitro* for 72 h and then viable cells were resuspended to 5 × 10^7^ cells in 50% PBS+ 50% Matrigel. Twenty 6-week old male C57BL/6 mice (RRID:MGI:2159769) were purchased from Charles River (Kent, UK) and housed in a controlled environment as described above. Mice were randomised into two groups to receive a single subcutaneous injection with 100 μL of TRAMPC2 cell suspension (5 × 10^6^ cells) in the left flank. Tumour progression was monitored weekly based on bioluminescence using the IVIS system for 6 weeks and the images were blinded for data analysis. Mice were euthanized through exsanguination under general anaesthesia, followed by cervical dislocation.

#### RM1 bone metastasis study

RM1 cells^37^ were firstly pre-treated with 256 μM P-SiaFNEtoc or vehicle (7.5% DMSO) *in vitro* for 72 h and then viable cells were resuspended in PBS to 1 × 10^6^ cells/mL. 6-week old male C57BL/6 mice (RRID:MGI:2159769) were purchased from Charles River (Kent, UK) and housed in a controlled environment as described above. Twenty mice were randomised into two groups to receive single-cell suspensions of 1 × 10^5^ RM1 (vehicle control or P-SiaFNEtoc pre-treated) cells/100 μL PBS via intracardiac injection. Tumour progression was monitored weekly based on bioluminescence using the IVIS system for 2 weeks and the images were blinded for data analysis. Mice were euthanized through exsanguination under general anaesthesia, followed by cervical dislocation.

#### Micro-CT analysis

Left tibias were dissected and scanned by SkyScan 1172 desktop micro-CT (Bruker, Massachusetts, USA) at the resolution of 4.3 μm. Trabecular bone volume fraction (BV/TV) and trabecular number (Tb.N) were measured from a 1.0 mm thick region 0.2 mm above the growth plate where metastatic tumour cells are generally situated. Nomenclature and symbols were used to describe the micro-CT derived bone morphometries according to the published guidelines.^38^

#### Bone histomorphometry

Left tibias were dissected and fixed in 10% buffered formaldehyde, decalcified in 14.3% EDTA, and then embedded in paraffin wax. Sections were cut longitudinally at 3 μm thickness and stained using a tartrate-resistant acid phosphatase (TRAP) staining kit (Sigma, 386A), according to the manufacturer’s protocol. The number of osteoblasts (N.Ob/B.Pm) and the number of osteoclasts (N.Oc/B.Pm) were determined on a 1.5 mm length of lateral and medial endocortical surfaces respectively, using a Leitz DMRB microscope (Leica Microsystems, Wetzlar, Germany). All histomorphometric nomenclature were based on the published guidelines^39^ and were obtained using the Osteomeasure bone histomorphometry software (OsteoMetrics, Inc. Decatur, GA, USA).

#### Generation of murine derived osteoclasts

Osteoclast precursors were isolated from the long bones of mice and osteoclast differentiation was performed in 50% conditioned media (prepared as described previously^22^) supplemented with M-CSF and RANKL as outlined previously.^40^ The successful formation of mature osteoclasts following treatment was determined by TRAP staining as described above. Osteoclasts were quantified in 5 fields of view at 10× magnification using ImageJ software.

#### Generation of human monocyte-derived macrophages

Leucocyte cones were ordered from the National Health Service Blood and Transplant Service (NHSBTS). Cells were mixed 1:1 with phosphate-buffered saline (PBS) and layered on Ficoll–Paque (GE Healthcare; 1714402). Cells were spun at 400 G for 30 min, with the brake off, and the human peripheral blood mononuclear cells were taken from the buffy layer above the Ficoll–Paque. CD14+ cells were isolated from peripheral blood mononuclear cells using the MACS system (Miltenyi Biotech; 130-050-201. LS Columns; 130-042-401). CD14+ cells were plated at 1 × 10^6^/mL in 850 mL AIM-V media (ThermoFisher; 12055091) plus 150 μl concentrated supernatant from wild type PC3 cells or PC3 cells overexpressing ST6GAL1 in the presence of either 5 μg/ml αM-CSF (Biolegend; 699203) or isotope control (Biolegend; 401215). Supernatant and antibodies were replenished every 3 days. Control cells were treated at 1 × 10^6^/ml in 1 ml AIM-V media supplemented with 50 ng/ml recombinant M-CSF (replenished every 3 days; Biolegend; 574804) in the presence of either 5 μg/ml αM-CSF or isotope control. All cells were cultured for 6 days before harvesting.

#### Flow cytometry (macrophages)

1 × 10^5^ cells were stained with a live/dead dye (ThermoFisher; L23102) in PBS for 10 min on ice in the dark, before being washed twice in FACS buffer (0.5% bovine serum albumin [Sigma; 05482] in PBS + 2 mM EDTA). Cells were then Fc blocked with Trustain (Biolegend; 422302) in FACS buffer for 10 min on ice in the dark. Cells were washed and then stained with anti-CD206-PerCP (Biolegend; 321121) or isotype control (Biolegend; 400149), using concentrations recommended by the manufacturer, on ice for 30 min in the dark. Cells were washed and read using a BD Accuri C6 Plus flow cytometer, with analysis carried out using BD Accuri C6 Plus software. All cells were gated as follows: (a) Forward scatter and side scatter (SSC) to exclude cellular debris (whilst also adjusting threshold), (b) live/dead (only live cells carried forward) and (c) SSC-A vs. SSC-H—only singlets carried forward. MFIs were corrected against the isotype control.

#### RNA sequencing analysis

RNA sequencing data can be accessed on the GEO repository (submission GSE236208). RNA was extracted from PC3 or DU145 cell lines transduced with negative-control lentiviral particles or with stable ST6GAL1 overexpression with 3 biological repeats per experimental condition. Samples were prepared as described previously^41^ and sequenced using an Illumina NextSeq 500, giving 15 million 75 bp single reads per sample. All data analyses were performed in Galaxy version 22.01.^42^ Quality control was performed with FastQC (http://www.bioinformatics.babraham.ac.uk/projects/fastqc/) and reads were trimmed with Cutadapt.^43^ Reads were mapped to hg38 using HISAT2^44^ and quantified with featureCounts.^45^ Differential gene expression analysis was performed using limma-voom^46^ and a volcano plot was generated with ggplot2.^47^ Gene ontology (GO) analysis was performed with goseq^48^ applying a significance threshold of adjusted p value < 0.05 for differentially expressed genes. Gene Set Enrichment Analysis (GSEA) was performed with the package EGSEA.^49^ Normalised count matrix values were used to create a heatmap with gplots.^50^

#### Western blotting

Western blotting was performed as previously described.^25^^,^^26^ Immunoblots were probed with antibodies for ST6GAL1 at 1:1000 dilution (Abgent, AP19891c), desmoplakin at 1:1000 (Thermo Fisher, A303-355A), actin at 1:2000 (Sigma, A2668, RRID:AB_258014) or GAPDH (Abgent, AP7873b) followed by incubation with appropriate HRP-conjugated secondary antibodies.

#### Immunocytochemistry

Cells were cultured in Lab-Tek™II Chamber Slides (Thermo Scientific, 154453) for 72 h in complete media. Cells were washed with PBS before permeabilization and fixation with ice-cold absolute methanol for 10 min at −20 °C. Next, slides were washed with PBS and blocked with 10% goat serum (Abcam, ab7481) for 1hr at room temperature. Slides were incubated overnight at 4 °C with ST6GAL1 at 1:200 (Abgent, AP19891c) or desmoplakin at 1:100 (Bethyl, A303-355A-T), followed by goat anti-rabbit IgG H&L (Alexa Fluor® 594) (Abcam, ab150080), diluted 1:500. Finally, slides were washed with PBS and stained with Hoechst (Thermo Scientific, 62249) for 15 min at room temperature. Images were acquired and processed with the ZEISS Axio Imager 5.

To profile Siglec ligands in human cell lines, chamber slides were washed in cold PBS-T, blocked in 1X Carbo-Free Blocking Solution (1X CFB) (Vector Laboratories, SP-5040-125) and labelled with Siglec-hFCs provided by Professor Matthew Macauley.^51^ Provided reagents were pre-complexed to streptavidin-AF647 (Abcam, ab272190) in the dark for 30 min at 4 °C. Cells were washed 3 times in PBS and incubated with Hoechst for nuclear staining. For neuraminidase treatments, α2-3,6,8 neuraminidase (NEB, P0720) was used as a negative control to strip sialic acid from the surface of cells. Cells were cultured in 100 units/mL neuraminidase in serum free conditions for 8 h at 37 °C in a humidified atmosphere containing 5% carbon dioxide. Images were obtained using a ZEISS Axio Imager 2 microscope. Siglec binding intensity was measured by analysing the fluorescent images using Fiji software^52^ by measuring the area, integrated density and mean grey value of one cell (n = 125). The fluorescence intensity was calculated in Excel using the formula for corrected total cell fluorescence (CTCF) = integrated density–(area of selected cell × mean fluorescence of background readings).

Siglec ligands in mouse cell lines were profiled using Recombinant Mouse Siglec-2/CD22 Fc Chimera Protein (C-terminus Human IgG1) (R&D Systems, 2296-SL) and Recombinant Mouse Siglec-3/CD33 Fc Chimera Protein (C-terminus Mouse IgG2a) (R&D Systems, 10102-SL) at 10 ng/μl. Cells were blocked in 1X CFB before incubation with recombinant Siglec-Fcs overnight at 4 °C. Cells were washed 3 times in PBS and incubated with ChromoTek Nano-Secondary alpaca anti-human IgG, recombinant VHH, CoraLite Plus 647 (Proteintech, shuGCL647-2) or ChromoTek Nano-Secondary alpaca anti-mouse IgG2a, recombinant VHH, CoraLite Plus 647 (Proteintech, shuGCL647-2) at 1 ng/μl in the dark for 1 h at room temperature. Nuclear staining and CTCF quantification (n = 200) were performed as above. Images were acquired with a ZEISS Axio Imager 5 microscope.

#### Lectin immunofluorescence

Cells were cultured in Lab-Tek™II Chamber Slides (Thermo Scientific, 154453) for 72 h in complete media containing DMSO (vehicle control) or 256 μM P-SiaFNEtoc. Cells were washed with PBS before permeabilization and fixation with ice-cold absolute methanol for 10 min at −20 °C. Next, slides were washed with PBS and blocked with 1X Carbo-Free™ Blocking Solution (1X CFB) (Vector Laboratories, SP-5040-125) for 1hr at room temperature. Slides were incubated for 3 h at room temperature with FITC-conjugated SNA lectin (Vector labs, FL-1301-2) at 1:500 or FITC-conjugated MAL I Lectin (Vector labs, FL-1311-2) at 1:500. Finally, slides were washed with PBS and stained with Hoechst (Thermo Scientific, 62249) for 15 min at room temperature. Cells were mounted using ProLong™ gold antifade mountant (Thermo Fisher, P36930). Images were acquired and processed with the ZEISS Axio Imager 4.

#### Flow cytometry using lectins and lectenz

Cells were cultured for 72 h in complete media containing DMSO (vehicle control) or the indicated concentrations of P-SiaFNEtoc. Cells were washed with PBS and harvested with trypsin and centrifugation (500×*g*, 5 min at room temperature). The cells were washed twice with 1X Carbo-Free Blocking Solution (1X CFB) (Vector labs, SP-5040-125) then resuspended in 100 μL of 1:2000 FITC-conjugated SNA lectin in 1X CFB and incubated for 30 min at 4 °C. Cells were washed with PBS twice before being resuspended in 500 μL PBS with 1 μg/mL propidium iodide. 10,000 events per sample were acquired on a BD LSRFortessa™ Cell Analyzer (BD Biosciences). Data was analysed using the FCS Express™ Flow Cytometry Analysis Software (the histograms shown are representative of three biological repeats). For the Lectenz staining, a solution of 2 μg/mL Pan-specific Lectenz (Lectenz bio, SK0501B) with 0.8 μg/mL Strep-PE in 1X CFB containing 1 mM CaCl_2_ and 1 mM MgCl_2_ was made for at least 10 min before use. The cells were harvested with trypsin and centrifugation (2000 rpm at 4 °C), washed with 100 μL PBS and then resuspended in 50 μL of the pre-incubated staining solution and incubated at 4 °C for 60 min. Cells were then washed three times with 100 μL PBA (PBS containing 1% v/v FBS and 0.02% w/w sodium azide) and resuspended in 50–100 μL PBA. Fluorescence was measured with a FACSCalibur (BD Biosciences) and a Cytoflex flow cytometer (Beckman & Coulter). Each replicate for each condition had >10,000 gated events. Data was processed using FlowJo V10 (FlowJo LLC). The percentage of lectin binding was obtained by normalizing to the median fluorescent intensity (MFI) values of the respective DMSO control.

#### Cell titre glo assays

Cell titre Glo assays were performed as described previously.^41^ Cell viability was assessed at 72 h with the CellTiter-Glo® Luminescent Cell Viability Assay (Promega, G7571) and luminescence was recorded with the Varioskan™ LUX microplate reader.

#### Statistical analyses

Statistical analyses were conducted using the GraphPad Prism software (version Prism 9.4.1). Data are presented as the mean of three independent samples ± standard error of the mean (SEM). Statistical significance is denoted as ∗p < 0.05, ∗∗p < 0.01, ∗∗∗p < 0.001 and ∗∗∗∗p < 0.0001.

#### Ethics

##### Clinical samples

The RNA samples analysed in Supplementary Fig. S1a were previously published.^27^^,^^53^ The RNA samples analysed in Supplementary Fig. S1b were kindly provided by the Prostate Cancer Biorepository Network (PCBN). The prostate tissue, rapid autopsy samples and serum samples used in this study were also provided by PCBN. Written informed consent was obtained from all patients. The PCBN ethics committee reviewed our project and provided ethical approval for use of the samples in our project (refs: 181029.1, 191112.1, 210412.1, 210203.1 and 200522.1).

##### Mouse models

All procedures complied with the UK Animals (Scientific Procedures) Act 1986 and were reviewed and approved by the local Research Ethics Committees of the University of Sheffield under Home Office project licence (PP3267943). The animal research performed in this study is reported in accordance with ARRIVE guidelines.

##### Human monocytes

Leucocyte cones were ordered from the National Health Service Blood and Transplant Service (NHSBTS). The NHSBTS obtains informed consent from donors to provide materials to partners under the terms of their UK HTA (Human Tissue Authority) licence. Partners must apply for access to the materials within the terms of this licence.

#### Reagent validation

The ST6GAL1 antibody used in this study has previously been published by us and validated for use in immunohistochemistry.^22^ The Siglec-Fc proteins are previously published,^51^ and confirming the specificity of our results, no specific binding was detected using mutated Siglec-Fc proteins,^51^ and binding was eliminated when cells were treated with neuraminidase. The cell lines used were validated via STR profiling and tested monthly for mycoplasma contamination.

#### Role of funders

The funders of this study did not play a role in the study design, data collection, data analyses, interpretation, or the writing of this manuscript.

## Results

### The sialyltransferase enzyme ST6GAL1 is upregulated in patients with prostate cancer that have bone metastasis

The sialyltransferase ST6GAL1 has been previously identified as upregulated in prostate cancer and linked with poor overall survival^20^ but a correlation between ST6GAL1 and prostate cancer metastasis has not yet been reported. To address this gap, we here monitored ST6GAL1 in five additional prostate cancer clinical cohorts. Using quantitative PCR, we analysed *ST6GAL1* gene levels in a molecular subgroup of patients with prostate cancer that have metastatic potential at presentation (previously published by^25^^,^^53^). Within this dataset, *ST6GAL1* is 6.3 -fold upregulated in the ‘metastatic’ subgroup compared to the ‘non-metastatic’ sub-group, suggesting upregulation of the *ST6GAL1* gene in patients with primary prostate cancer presenting with metastatic biology (n = 20, unpaired t test, p = 0.024) (Supplementary Fig. S1a). Furthermore, *ST6GAL1* gene levels are 2.1-fold higher in castrate-resistant prostate cancer (CRPC) compared to hormone naïve disease (n = 20, unpaired t test, p = 0.0023) (Supplementary Fig. S1b). Next, using immunohistochemistry (IHC) we analysed ST6GAL1 levels in a 96 case tissue microarray (TMA) comprising tissue from patients with prostate cancer diagnosed with tumours of different Gleason grades.^26^ Our findings show patients with Gleason grade 7–10 prostate cancerhave 2-fold higher ST6GAL1 levels than patients diagnosed with Gleason 6 tumours (unpaired t test, p = 0.0042) (Supplementary Fig. S1c). We did not detect a significant difference in ST6GAL1 expression between Gleason 7–8 and Gleason 8–10 tumours in this TMA (unpaired t test, p = 0.4542). Next, we analysed a second 125 case TMA^29^ to compare ST6GAL1 levels in localised prostate cancer tumours and prostate tumours presenting with metastasis to bone or lymph node (all biopsy samples were taken from the primary site) (Supplementary Table S1). Our findings suggest ST6GAL1 is 2.3-fold upregulated in patients with prostate cancer bone metastasis compared to patients with localised disease (unpaired t test, p = 0.0091) (Fig. 1a). Taken together, our data suggests ST6GAL1 may be upregulated in patients with prostate cancer that have increased risk of metastasis, those developing relapse to castrate-resistant disease, and patients with prostate tumours that have spread to the bone.

### ST6GAL1 is upregulated in prostate-derived tumours growing in bone

The above data shows ST6GAL1 levels are significantly increased in prostate tumours from patients with cancer that has spread to bone. To investigate if prostate-derived tumours growing in bone also have high levels of ST6GAL1 we analysed 20 rapid autopsy samples taken from men with bone metastasis (these samples were from prostate-derived tumours growing in bone and were obtained within hours of death). As these sections were stained at the same time as the TMA shown in Fig. 1a, this enabled us to compare ST6GAL1 levels in prostate-derived tumours growing in bone to primary prostate cancer tissue. Our data shows ST6GAL1 is 1.7-fold higher in prostate-derived tumours growing in bone compared to tumours biopsied at the primary site (unpaired t test, p = 0.0103) (Fig. 2a). Using sialic acid isomer stabilization,^33^ it is possible to specifically detect α2-6 linked sialic acids (which have a +27 mass shift and are easy to differentiate from α2-3 linked sialic acids that instead have a +37 mass shift). We previously showed larger branched *N-*glycans with α2-6 sialylation, including α2-6 sialylated tri-antennary and tetra-antennary glycans, are common to primary prostate tumours.^22^^,^^33^ Next, following detection of ST6GAL1 in rapid autopsy bone metastasis tissue by IHC, the same tissue cores were processed for MALDI imaging mass spectrometry (MALDI-IMS) of *N*-glycans.^22^^,^^33^^,^^54^ We detected *N*-glycans with both α2-6 and α2-3 linked sialic acids in all 20 prostate cancer bone metastasis tissues. Representative α2-6-linked sialylated *N*-glycans from three tissues with high ST6GAL1 levels are shown (Fig. 2b). These findings, taken together with previous studies,^22^^,^^33^ show that like primary prostate tumour tissue, prostate-derived tumours growing in bone also contain an abundance of single branched α2-6 sialylated tri-antennary and tetra-antennary *N*-glycans. However, more tri-antennary sialylated *N*-glycans are represented in bone metastatic tissue, with a notable increase in tissues with two or more α2-6 sialylated species. Of particular interest, unique single α2-6 sialylated poly-lactosamine containing *N*-glycans were also detected, which have been associated with poor clinical outcomes in metastatic breast cancers.^55^ Together, our data suggests that levels of both ST6GAL1 and larger branched α2-6 sialylated tri-antennary and tetra-antennary *N*-glycans remain high in prostate cancer cells after they have disseminated from the primary site and established metastatic lesions in bone.

### Levels of blood borne ST6GAL1 are increased in men with aggressive prostate cancer

We recently showed ST6GAL1 is upregulated in blood samples from men with prostate cancer, compared to men with either benign disease or men given a ‘no cancer’ diagnosis.^22^ Our findings above suggest the levels of ST6GAL1 are also upregulated in aggressive prostate tumour tissue, however it is unclear whether the levels of blood borne ST6GAL1 are also higher in men with more aggressive disease. To test this, we used pre-validated sandwich ELISA assays^22^ to monitor ST6GAL1 protein levels in a cohort of 300 serum samples, including 40 men given a ‘no cancer diagnosis’, 100 men with low grade prostate cancer (Gleason grade 6–7), 100 men with high grade prostate cancer (Gleason grade 8–9), and 60 men with metastatic CRPC (Supplementary Table S1). In agreement with our previous findings,^22^ ST6GAL1 serum levels were 7.3-fold higher in men diagnosed with prostate cancer relative to men without prostate cancer (n = 240, unpaired t test, p < 0.0001) (Fig. 3a). When serum ST6GAL1 levels were compared in men with low grade and high grade prostate cancer (Gleason grade 6–7 compared to Gleason grade 8–9), levels of ST6GAL1 were found to be 6.7-fold higher in men with higher grade compared to men with lower grade disease (n = 200, unpaired t test, p < 0.0001) (Fig. 3b). Similarly, serum ST6GAL1 was 10.6-fold higher in men with clinically significant prostate cancer compared to non-clinically significant prostate cancer defined according to the PI-RADS v2.1 guidelines^30^ (n = 200, unpaired t test, p = 0.143) (Fig. 3c) and levels of serum ST6GAL1 were 4.4-fold higher in men with a prostate tumour volume ≥0.5 cc compared to those with a tumour volume <0.5 cc (where a tumour volume of ≥0.5 cc is defined as clinically significant)^31^^,^^32^ (n = 200, unpaired t test, p = 0.0174) (Fig. 3d) and 1.55-fold higher in prostate tumours extending outside the prostate gland (capsular perforation) compared to tumours not involving or extending beyond the prostate gland (n = 200, unpaired t test, p = 0.0394) (Fig. 3e). Finally, levels of serum ST6GAL1 were 2.24-fold higher in men with metastatic CRPC (58/60 of whom had metastasis to bone) compared to men with hormone treatment naïve disease (n = 260, unpaired t test, p = 0.0460) (Fig. 3f). In summary, our findings confirm blood borne ST6GAL1 levels are increased in men diagnosed with prostate cancer, and further show levels of ST6GAL1 are significantly higher in the blood of men with more aggressive prostate tumours, and upon relapse to metastatic castrate resistant disease.

### Upregulation of ST6GAL1 promotes the spread of prostate cancer to bone and modulates the pre-metastatic niche

**Fig. 4:**
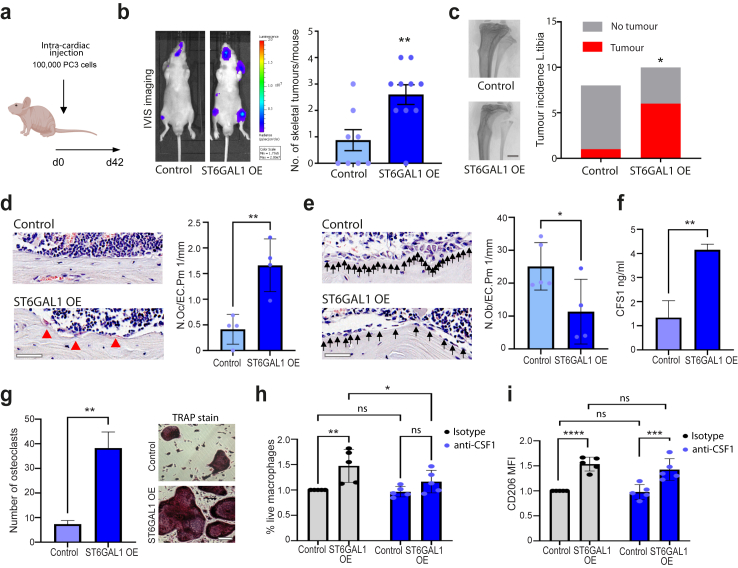
Upregulation of ST6GAL1 promotes the spread of prostate cancer to bone. (**a**) Luciferase tagged PC3 cells (control or ST6GAL1 overexpressing) were injected into BALB/c nude mice via intra cardiac injection. (**b**) Tumours were monitored over 6 weeks using *in vivo* bioluminescence imaging. Upregulation of ST6GAL1 significantly increased the number of skeletal tumours formed per mouse (n = 10, unpaired t test, p = 0.006). (**c**) *Ex vivo* micro-CT analysis was used to examine tumour induced bone destruction in left tibias and showed that overexpression of ST6GAL1 in PC3 cells significantly enhanced tumour incidence (representative images are shown) (n = 10, Chi-square test, p = 0.04). Scale bar is 1 mm. (**d**,**e**) Further bone histomorphometry demonstrated that in mice bearing ST6GAL1 overexpressing tumours there were significantly higher number of osteoclasts per mm medial bone surface (n = 4 unpaired t test, p = 0.0055) but lower number of osteoblasts per mm lateral bone surface in tibias without overt tumours, compared to mice bearing control tumours (n = 5 unpaired t test, p = 0.0452). (**f**) Sandwich ELISA analysis of CSF1 levels in conditioned media samples from PC3 prostate cancer cells with overexpression of ST6GAL1. CSF1 levels are significantly increased in conditioned media samples from PC3 cells with upregulation of ST6GAL1 (unpaired t test, p = 0.003). (**g**) Conditioned media samples from DU145 cells overexpressing ST6GAL1 promotes the differentiation of murine primary osteoclast pre-cursors into osteoclasts measured via TRAP staining (unpaired t test, p = 0.0005). Scale bar is 100 μm. (**h**,**i**) Primary monocytes from healthy controls were treated with 150 μl of concentrated conditioned media from PC3 cells (control or ST6GAL1 overexpressing) at days 0 and 3 in the presence of 5 μg/ml anti-MCSF or isotype control. Cells were harvested on day 6 and assessed for viability (h) and CD206 expression (i) using flow cytometry. (n = 5, two-way ANOVA, multiple comparisons, (h) cont. vs. OE isotype. p = 0.002. OE isotype vs. OE anti-MCSF p = 0.028. (i) cont. vs OE isotype. p < 0.0001. cont. vs OE isotype. p = 0.0002).

We previously showed that ST6GAL1-mediated aberrant sialylation increases the growth of prostate tumours and is linked to disease progression.^22^ However, to date the *in vivo* role of ST6GAL1 in prostate cancer metastasis has not yet been investigated. As the above data indicates that ST6GAL1 is specifically upregulated in patients with prostate cancer bone metastasis, we chose to investigate whether ST6GAL1 plays a functional role in the spread of prostate cancer to bone. Using lentiviral transductions, we created luciferase labelled PC3 cells with upregulation of the ST6GAL1 enzyme, as PC3 has the lowest level of endogenous ST6GAL1 (Supplementary Fig. S2). PC3 cells are human metastatic prostate cancer cells that were originally established from skeletal metastases and preferentially form tumours in bone.^56^ As expected, PC3 cells overexpressing ST6GAL1 had significantly higher levels of α2-6 sialylated *N*-glycans (detected using SNA lectin^57^) (Supplementary Fig. S2). Next, using an intra-cardiac injection immunocompromised mouse model, we found that upregulation of ST6GAL1 significantly increased the number of metastatic tumours formed by PC3 cells in bone (unpaired t test, p = 0.006) (Fig. 4a and b). Furthermore, *ex vivo* micro-CT analysis revealed that overexpression of ST6GAL1 significantly enhanced tumour incidence in long bones and associated bone destruction (left tibias, Chi-square, p = 0.04) (Fig. 4c and Supplementary Fig. S3). Consistent with enhanced bone destruction, using histomorphometry, we detected an increased presence of bone resorbing osteoclasts and a decreased number of bone forming osteoblasts on endocortical bone surfaces (Supplementary Fig. S3). More importantly, when compared to mice bearing control tumours, mice bearing ST6GAL1 overexpressing tumours had a 4.02-fold increase in the number of osteoclasts (unpaired t test, p = 0.0055) (Fig. 4d) and a 55% reduction in the number of osteoblasts on endocortical surfaces in tibias without overt tumours (unpaired t test, p = 0.0452) (Fig. 4e). This data raises the possibility that upregulation of ST6GAL1 in tumours in sites beyond bones could modify the bone pre-metastatic niche towards bone resorption to facilitate the initiation of the vicious cycle.

Previous studies have shown ST6GAL1-mediated α2-6 sialylation impacts cancer hallmarks and regulates oncogenic cell behaviours, however the specific targets and signalling pathways orchestrated by ST6GAL1 require greater delineation.^17^ Next, to investigate how ST6GAL1 changes prostate cancer cell behaviour towards a more metastatic biology, we used RNA-sequencing (RNA-Seq) of PC3 and DU145 prostate cancer cells. When ST6GAL1 is overexpressed, bioinformatic analyses identified 776 differentially expressed genes in PC3 cells, and 1156 differentially expressed genes in DU145 cells (adjusted p value < 0.05, Log_2_FC 0.58) (Supplementary Fig. S4 and S5 and Tables S2 and S3). Gene set enrichment analysis revealed PC3 cells overexpressing ST6GAL1 have upregulation in ‘interferon alpha response’, ‘complement system’ and downregulation in ‘epithelial mesenchymal transition’ (EMT) and ‘inflammatory response’ (Supplementary Fig. S4). DU145 cells overexpressing ST6GAL1 had upregulation in the ‘epithelial mesenchymal transition (EMT)’, ‘inflammatory response’ and ‘IL6 JAK STAT3 signalling’ hallmark signatures (Supplementary Fig. S5). ST6GAL1 also correlated with increased expression of inhibitor of DNA binding 2 (ID2) which is implicated in tumour metastasis,^58^ downregulation of the tumour suppressor protein desmoplakin (DSP),^59^ and increased secretion of insulin like growth factor binding protein 5 (IGFBP5) which is implicated in prostate cancer progression^60^ (Supplementary Fig. S6). Of particular interest, we also detected increased secretion of colony-stimulating factor-1 (CSF1) in prostate cancer cells overexpressing ST6GAL1 (Fig. 4f and Supplementary Fig. S6).

CSF1, also known as macrophage colony stimulating factor (MCSF), is crucial for the function and differentiation of myeloid lineage cells including osteoclasts and monocytes/macrophages^61^ and is implicated in sustaining the functions of tumour-associated macrophages (TAMs) and resistance to immune checkpoint blockade.^62^, ^63^, ^64^, ^65^ As ST6GAL1 overexpressing prostate cancer cells have increased secretion of CSF1, we next used co-culture models to assess the potential effects of prostate cancer cells on osteoclasts and macrophages. Our findings show that conditioned media from prostate cancer cells overexpressing ST6GAL1 cells can promote primary murine monocyte/macrophage like cells to differentiate into osteoclasts (unpaired t test, p = 0.0013) (Fig. 4g). Upregulation of ST6GAL1 also significantly enhanced the development of primary human monocytes into macrophages (Two-way ANOVA, p = 0.002) and this was dependent upon CSF1 (Two-way ANOVA, p = 0.0278) (Fig. 4h). In prostate cancer, TAMs characterised by an immunosuppressive M2 phenotype represent a major component of the tumour microenvironment (TME).^66^, ^67^, ^68^, ^69^ As prostate tumours are infiltrated with CD206 positive M2 macrophages and this correlates with progression to metastatic disease,^70^ we assessed the effects of ST6GAL1 overexpressing prostate cancer cells on human macrophages using a co-culture study. Excitingly, our data shows that upregulation of ST6GAL1 in prostate cancer cells significantly increases expression of CD206 on macrophages, suggesting a shift toward a more immunosuppressive M2 phenotype (Two-way ANOVA, p < 0.0001) which was not dependent upon CSF1 (Fig. 4i). Taken together, our findings reveal that ST6GAL1-mediated aberrant sialylation plays a role in the spread of prostate cancer to bone and suggests upregulation of ST6GAL1 in prostate cancer cells can modulate the pre-metastatic niche by enhancing bone destruction to encourage the outgrowth of incoming prostate cancer cells. Furthermore, factors secreted by ST6GAL1 overexpressing prostate cancer cells may promote the development of TAMs and contribute to an immunosuppressive TME.

### ST6GAL1 regulates immunosuppressive sialoglycans in prostate cancer cells

**Fig. 5:**
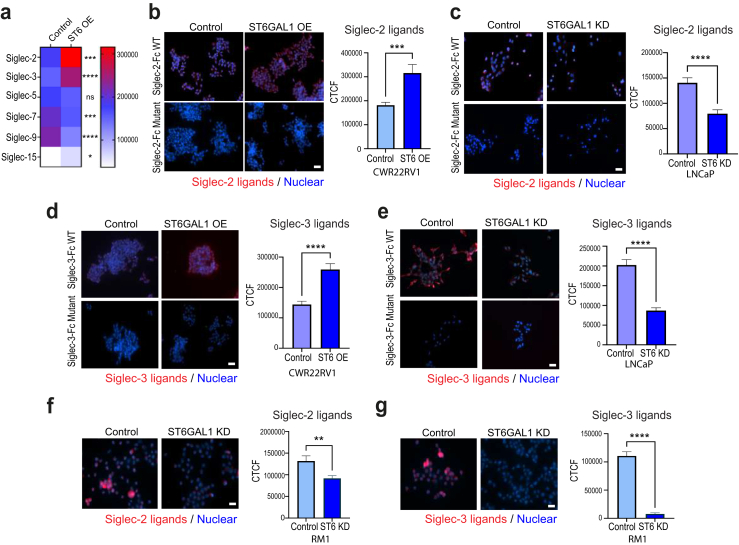
ST6GAL1 regulates immunosuppressive sialoglycans in prostate cancer cells. Siglec ligands were monitored in prostate cancer cells with immunocytochemistry using recently developed Siglec-Fc proteins.^51^ (**a**) Heatmap to illustrate Siglec ligands that are differentially expressed in CWR22RV1 prostate cancer cells with overexpression of ST6GAL1. (**b**) Upregulation of ST6GAL1 in CWR22RV1 cells significantly increases sialoglycans that engage Siglec-2 (CD-22) (unpaired t test, p = 0.0008). (**c**) Downregulation of ST6GAL1 in LNCaP prostate cancer cells significantly reduced the expression of sialoglycans that engage Siglec-2 (unpaired t test, p < 0.0001). (**d**) Upregulation of ST6GAL1 in CWR22RV1 cells significantly increases the levels of sialoglycans that engage Siglec-3 (CD33) (unpaired t test, p < 0.0001). (**e**) Downregulation of ST6GAL1 in LNCaP prostate cancer cells significantly reduced the expression of sialoglycans that engage Siglec-3 (unpaired t test, p < 0.0001). Scale bar is 50 μm. (**f**) Consistent with ST6GAL1 regulating ligands that are recognised by Siglec-2 and Siglec-3, we also detected downregulation of sialoglycans recognised by Siglec-2 (unpaired t test, p = 0.0036) and (**g**) Siglec-3 (unpaired t test, p = 0001) in murine RM1 prostate cancer cells depleted of ST6GAL1. Scale bar is 50 μm.

Siglecs (sialic acid-recognising Ig-superfamily lectins) are transmembrane sialoglycan binding proteins expressed on various immune cells.^7^ The tumour ‘glyco-code’ consists of numerous sialoglycans binding to Siglecs and the Siglec-sialoglycan axis plays a crucial role in tumour immune evasion.^71^ To investigate if ST6GAL1 can regulate immunosuppressive sialoglycans in prostate cancer cells, we utilised recently developed Siglec-Fc proteins^51^ to monitor Siglec ligands in CWR22RV1 prostate cancer cells with upregulation of ST6GAL1, and LNCaP cells with downregulation of ST6GAL1. Our findings reveal expression of ST6GAL1 alters sialoglycan patterns in prostate cancer cells, and in line with the literature,^72^^,^^73^ we find ST6GAL1 specifically regulates sialoglycans that engage Siglec-2 (CD22) and Siglec 3 (CD33) (Fig. 5a–e and Supplementary Fig. S7). Confirming the specificity of our results, no specific binding was detected using mutated Siglec-Fc proteins,^51^ and binding was eliminated when cells were treated with neuraminidase (which removes terminal sialic acids of glycans) (Supplementary Fig. S7). Consistent with ST6GAL1 regulating Siglec-2 and Siglec-3 ligands, we also detected downregulation of sialoglycans that engage Siglec-2 and Siglec-3 in murine RM1 prostate cancer cells depleted of ST6GAL1 (Fig. 5f and g). Together with the findings above, these data suggest the mechanisms underlying the role of ST6GAL1 in prostate cancer are multi-faceted, and also likely involve the regulation of immunosuppressive sialoglycans that can engage immune cells.

### Sialic acid blockade can prevent/inhibit prostate cancer bone metastasis

**Fig. 6:**
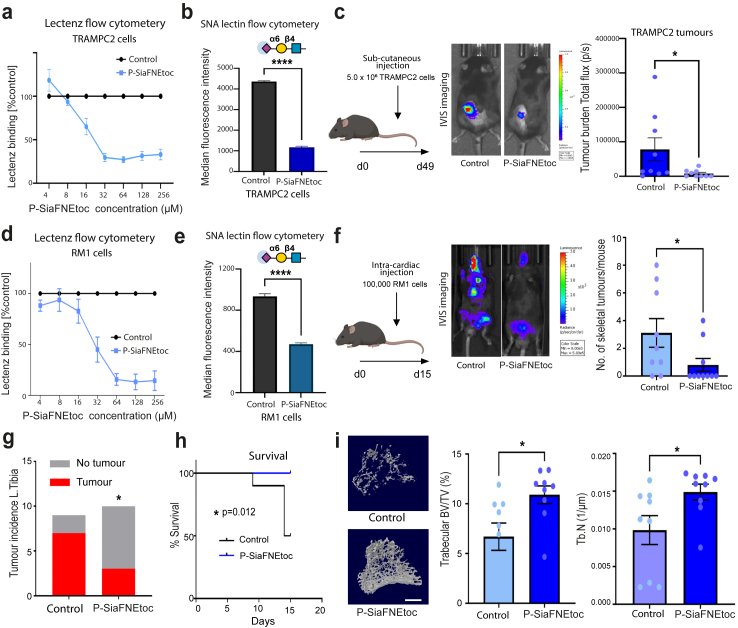
Sialic acid blockade can prevent/inhibit prostate cancer bone metastasis. (**a**) Inhibition of sialylation in TRAMPC2 cells using P-SiaFNEtoc detected using pan-specific Lectenz lectin flow cytometry.^74^ Cells were treated with a range of concentrations of P-SiaFNEtoc inhibitor from 2 μM to 512 μM for 72 h. The intensities were normalised to a DMSO control. (**b**) Detection of α2-6 linked sialylated *N*-glycans in TRAMPC2 cells using SNA lectin flow cytometry. TRAMPC2 cells treated with 64 μM P-SiaFNEtoc for 72 h had reduced levels of SNA binding indicating a reduction in α2-6 linked sialylation in these cells (unpaired t test, p = 0.0001). (**c**) Luciferase tagged TRAMPC2 cells (control or pre-treated with 64 μM P-SiaFNEtoc for 72 h) were injected into immunocompetent C57BL/6 mice via sub-cutaneous injection and tumours were monitored using *in vivo* bioluminescence imaging. Pre-treatment of TRAMPC2 cells with P-SiaFNEtoc (which removed sialylated glycans) significantly reduced tumour burden over 6 weeks (n = 10, Mann–Whitney test, p = 0.0233) thus suggesting that sialic acid blockade has the potential to inhibit the growth of prostate tumours. (**d**) Inhibition of sialylation in RM1 cells using P-SiaFNEtoc detected using pan-specific Lectenz lectin flow cytometry.^74^ Cells were treated with a range of concentrations of P-SiaFNEtoc inhibitor from 2 μM to 512 μM for 72 h. The intensities were normalised to a DMSO control. (**e**) Detection of α2-6 linked sialylated *N*-glycans in RM1 cells using SNA lectin flow cytometry. RM1 cells treated with 256 μM P-SiaFNEtoc for 72 h had reduced levels of SNA binding indicating a reduction in α2-6 linked sialylation in these cells (unpaired t test, p < 0.0001). (**f**) Luciferase tagged RM1 cells (control or pre-treated with 256 μM P-SiaFNEtoc for 72 h) were injected into immunocompetent C57BL/6 mice via intra cardiac injection. Tumours were monitored over 15 days using *in vivo* bioluminescence imaging. (**g**,**h**) Pre-treatment of RM1 cells with P-SiaFNEtoc (to remove sialylated glycans) significantly reduced the number of skeletal tumours formed (Mann–Whitney test, p = 0.0454), the incidence of tumour in left tibias (Chi-square test, p = 0.0455), and significantly increased survival time in mice (Log-rank test, p = 0.012). (**i**) Micro-CT analysis demonstrated that P-SiaFNEtoc significantly alleviated bone destruction in the trabecular bone of tibias and increased trabecular bone volume (BV/TV, p = 0.0211) and trabecular number (Tb. N, p = 0.035) (n = 9, unpaired t test, ∗p < 0.05). Representative images are shown. Scale bar is 200 μm.

Taken together with previous findings,^22^ the above data indicate ST6GAL1 and α2,6 sialylated *N*-glycans play a functional role in prostate cancer progression and specifically in the spread of prostate tumours to bone. Previously, we used *in vitro* models to show that sialyltransferase inhibitors can be used to inhibit the α2,6 sialylation of prostate cancer cells with only minor side effects on other glycan types.^22^^,^^41^ However, to date the potential to utilise sialic acid blockade to inhibit the progression of prostate cancer has not yet been investigated using *in vivo* models. As sialoglycans can play crucial roles in tumour immune evasion,^7^^,^^71^ and our findings above indicate ST6GAL1 can regulate Siglecs ligands in prostate cancer cells, we next utilised syngeneic models^36^^,^^37^ to investigate if sialic acid blockade in mice with a functional immune system can suppress prostate tumour growth and bone metastatic prostate cancer. Our findings show treatment of murine TRAMPC2 prostate cancer cells with the sialylation inhibitor P-SiaFNEtoc^23^ inhibits α2,6 sialylation without impacting cell viability (Fig. 6a–b and Supplementary Fig. S8). When TRAMPC2 cells were pre-treated with P-SiaFNEtoc, before sub-cutaneous injection into immunocompetent C57BL6 mice, overt tumour burden was significantly reduced (n = 10, Mann–Whitney test, p = 0.0233) (Fig. 6c), thus suggesting that sialic acid blockade has the potential to inhibit the colonisation ability of prostate cancer cells.

The formation of secondary tumours in the bone is the most commonly observed site for prostate cancer metastasis and is significantly associated with reduced survival in patients.^3^ Next, we pre-treated murine RM1 prostate cancer cells with P-SiaFNEtoc to inhibit sialylation (Fig. 6d,e and Supplementary Fig. S8) before intra-cardiac injection into immunocompetent C57BL6 mice. Strikingly, sialic acid blockade with P-SiaFNEtoc significantly reduced the number of skeletal tumours formed by RM1 cells (Mann–Whitney test, p = 0.045) (Fig. 6f), the incidence of tumours in long bones (left tibias, Chi square test, p = 0.046 (Fig. 6g) and importantly also significantly increased the survival times of mice (Log-rank test, p = 0.012) (Fig. 6h). Further *ex vivo* micro-CT analysis demonstrated that de-sialylation significantly alleviated bone destruction and increased trabecular bone volume (BV/TV) and trabecular number (Tb. N) by 63% (n = 9, unpaired t test, p = 0.0211) and 51% (n = 9, unpaired t test, p = 0.035) (Fig. 6i). Taken together, these findings show that inhibiting sialylation in prostate cancer cells prevents/inhibits prostate cancer bone metastasis and provides proof of concept data that sialic acid blockade could be developed therapeutically for advanced prostate cancer.

## Discussion

Aberrant glycosylation is a hallmark of cancer and is not just a consequence, but also a driver of a malignant phenotype.^6^ In prostate cancer, changes to sialylated glycans are common and this has important implications for tumour growth, metastasis, and immune evasion.^75^^,^^76^ ST6GAL1 is an important sialyltransferase enzyme which is upregulated in many tumour types and plays a key role in cancer progression.^10^^,^^17^ Here, we find ST6GAL1 is upregulated in blood and tissue samples from patients with prostate cancer that have tumours which have spread to the bone and show that ST6GAL1 can promote prostate cancer metastasis *in vivo*. Furthermore, we reveal the mechanisms underlying how ST6GAL1 promotes bone metastasis likely involve the modification of the pre-metastatic niche towards bone resorption, promoting the development of M2 like immunosuppressive macrophages, and the regulation of immunosuppressive sialoglycans. Importantly, our study also provides proof-of-concept data to show that sialic acid blockade can inhibit prostate cancer bone metastasis and highlights the targeting of aberrant sialylation in combination with existing treatments as an exciting new strategy to develop novel combination therapies for patients with advanced prostate cancer.

Before metastasis occurs, the primary tumour can induce the formation of microenvironments in distant organs that are conductive to the survival and outgrowth of tumour cells – these microenvironments are termed pre-metastatic niches.^77^ In bone, the bone resorbing osteoclast is a major player for the formation of the pre-metastatic niche^78^ via modifying the bone matrix to facilitate the initiation of the ‘bone and cancer vicious cycle’.^79^^,^^80^ Although prostate cancer predominantly produces osteoblastic lesions, accelerated bone resorption or osteolysis is essential to the release of growth factors from the bone matrix which in turn initiate and stimulate metastatic cancer cell growth.^81^ Our *in vivo* findings demonstrate significantly higher number of osteoclasts in non-tumour bearing tibias in mice that have ST6GAL1 overexpressing tumours in sites beyond bones. This suggests upregulation of ST6GAL1 can modulate the bone pre-metastatic niche before metastatic spread takes place to induce bone resorption and potentially facilitate the initiation of the vicious cycle. It is tempting to propose that this modification of the pre-metastatic niche by prostate cancer cells is due to factors secreted by ST6GAL1 overexpressing cells (including CSF1), the ability of extracellular ST6GAL1 to modify sialylation and cell signalling pathways in recipient cells in the pre-metastatic niche, or a combination of these functions. Previous studies have indeed reported that cancer cells, including prostate cancer cells, can release functional ST6GAL1^22^^,^^82^ and our clinical data further demonstrates increased blood borne ST6GAL1 in men with aggressive prostate cancer. Combining this evidence with our data showing that ST6GAL1 can directly and indirectly regulate the differentiation and development of not only osteoclasts but one of its precursors - macrophage, it is very plausible that ST6GAL1 and/or aberrant α2-6 *N*-linked sialylation are involved in the formation of a pre-metastatic niche in bone.

In addition to promoting osteoclasts in the pre-metastatic niche, our data also suggests that ST6GAL1 enhances the development of M2 like macrophages and can also regulate sialoglycans on prostate cancer cells that engage Siglec receptors. Recent studies suggest a model whereby TAMs reside along a spectrum and not in mutually exclusive M1 or M2 polarisation states^83^, ^84^, ^85^ and it is clearly demonstrated that M2 macrophages are more effectively differentiated into osteoclasts.^86^ In prostate cancer, a higher density of macrophages in tumours is linked to poorer prognosis.^70^ Numbers of CD206+ M2 macrophages are increased in castrate resistant disease^70^^,^^87^, ^88^, ^89^ and consistent with our findings for ST6GAL1, a recent study showed CD206+ M2 macrophages are upregulated in bone metastatic CRPC specimens compared with primary tumours or lymph node metastases.^90^ M2 like macrophages can secrete pro-metastatic factors and cytokines to suppress immune responses initiated by T cell leading to immunological silence^91^ and strategies to target prostate tumour TAMs are being investigated as approaches to re-sensitise the prostate TME to immunotherapy.^66^^,^^92^ Previous studies have implicated tumour-derived sialic acids with the differentiation of monocytes to macrophages with a pathogenic phenotype.^93^^,^^94^ The findings presented suggest that upregulation of ST6GAL1 in prostate cancer cells could promote CD206+ M2 macrophages which could contribute to the relative resistance of bone metastatic CRPC to immune checkpoint therapy. Our study provides a platform for further mechanistic studies investigating the impact of ST6GAL1 and CD206+ macrophages in the prostate tumour immunosuppressive TME (for example in relation to T cell proliferation) and raises the possibility of enhancing therapies targeting prostate TAMs by concurrently blocking aberrant sialylation.

The specific glycan structures found on tumour cells, known as the tumour glyco-code, can alter how the immune system perceives cancer cells and can induce immune suppression.^95^ Siglec receptors, expressed by immune cells, are key mediators of this effect^71^^,^^96^ and the Siglec-sialoglycan axis can modulate immune cell function to promote an immunosuppressive microenvironment.^71^ The data presented reveals ST6GAL1 controls the expression sialoglycans on prostate cancer cells including ligands that could engage Siglec-2 (CD22) and Siglec-3 (CD33) on immune cells. Based on this, it is tempting to propose an additional mechanism underlying the role of ST6GAL1 in prostate cancer – the regulation of immunosuppressive sialylated glycans. Siglec-2 is primarily expressed on B cells,^97^^,^^98^ and while emerging evidence has identified tumour associated B cell responses in prostate cancer,^99^, ^100^, ^101^ a potential role of Siglec-2 in the prostate TME and specifically in bone metastasis is unclear. Siglec-3 is a myeloid cell marker^102^ that is being investigated as a therapeutic target for acute myeloid leukaemia.^103^ Siglec-3 is upregulated in castrate resistant prostate cancer^104^ and has previously been detected in bone metastatic prostate tumours.^105^ Preliminary findings presented here suggest that Siglec-3 ligands are expressed in prostate tumour tissue (Supplementary Fig. S7) suggesting that further studies investigating role of the Siglec-3 sialoglycan axis in the prostate cancer disease progression would be beneficial.

Strategies to inhibit aberrant sialylation in cancer are under development for a range of tumour types and include sialylation inhibitors, antibody-sialidase conjugates, Selectin inhibitors, and anti-Siglec antibodies.^7^^,^^12^ Sialytransferase inhibitors have been administered via intra-tumoural injection to suppress tumour growth^106^ and via targeted delivery using nanoparticles to block metastasis.^107^ However, systemic delivery of P-3F_AX_-Neu5Ac induces renal toxicity in mice, highlighting the need for better tolerated sialyltransferase inhibitors.^12^^,^^108^ Recently, C-5-modified 3-fluoro sialic acid sialyltransferase inhibitors, including P-SiaFNEtoc, have been developed which reach higher effective concentrations within the cell and hold potential to be used systemically.^23^^,^^24^ We previously showed that P-SiaFNEtoc can be used to specifically inhibit the sialylation of prostate with no side effects on other glycans.^41^ Here, we show that pre-treatment of prostate cancer cells with P-SiaFNEtoc can inhibit the growth of prostate tumours and the spread of prostate cancer to bone in syngeneic mouse models. Hypersialylation is a new immune checkpoint^109^ and a combination therapy combining desialylation and immune checkpoint blockade is currently in clinical trials for other cancer types (NCT05259696).^110^^,^^111^ In prostate cancer, we recently showed that anti-androgen treatments may inadvertently upregulate immunosuppressive sialoglycans suggesting that therapies to block sialylation may sensitise patients with prostate cancer to enzalutamide.^112^ Similarly, CSF1 signalling has been investigated as a target to reverse macrophage mediated resistance to androgen blockade therapy.^113^ Our study provides proof-of-principle data to demonstrate the efficacy of sialylation inhibition to block prostate tumour growth and bone metastasis and moving forward, we propose that sialic acid blockade should be explored in combination with existing anti-androgen and immunotherapy treatments to develop new urgently needed therapies for bone metastatic prostate cancer.

In conclusion, here we show that the sialyltransferase enzyme ST6GAL1 is upregulated in tumours and blood samples from patients with prostate cancer bone metastasis and that ST6GAL1-mediated aberrant sialylation can promote the spread of prostate tumours to bone. Our findings reveal the underlying mechanisms are multi-faceted and involve modification of the pre-metastatic niche towards bone resorption, the promotion of macrophage development and a shift towards a more M2 like phenotype, and the regulation of immunosuppressive sialoglycans. It is evident that ST6GAL1-mediated sialylation plays a central role in prostate cancer tumour pathology and metastasis, and thus holds huge potential for the development of new therapeutics. Our study provides proof-of-principle data to show that sialic acid blockade can be used inhibit the spread of prostate tumours to bone, and points to the need for further development in this area.

## Contributors

KH, EAG, MOM, ES, RG, RB, KL, EAV, JFAP, NE, SJM, ER, GH, RK, ZP, KPN, ENS, and EPD performed *in vitro* experiments. NW, MF, YZ, and MAL performed the *in vivo* studies. LW performed IHC on tissue sections. HT scored pathology sections. RRD performed and analysed N-glycan MALDI-IMS. KH, MOM and AD performed bioinformatic analyses. JM, KH, MOM, EAG, NW, MF, RG, ZP and RRD verified the underlying data.

JM and NW jointly designed, analysed and interpreted the study, wrote the original manuscript draft, made the figures, and are joint senior and corresponding authors. RRD, DJE, MM, JFAP, ER, CB, and TJB contributed to experiment design, and critical review of the manuscript. JM, NW, DJE, JFAP, ER, CB, ES, TJB, and RH contributed to funding acquisition. JM, NW, DJE, ES, MM, JFAP, ER, CB, TJB, and RH contributed to project supervision. All authors agree with the content of the manuscriptand were given the opportunity to provide input. All authors read and approved the final version of the manuscript.

## Data sharing statement

The authors confirm that the data supporting the findings of this study are available within the article and its Supplementary materials.

## Declaration of interests

JM and ES are shareholders of GlycoScoreDx Ltd and have filed patent applications related to this work (GB patent numbers GB2,594,103, GB2,595,425 and US patent application 17/780,508). J.F.A.P., N.E., E.R. are shareholders of and employed by GlycoTherapeutics B.V.; C.B. and T.J.B. are shareholders of and scientific advisors of GlycoTherapeutics B.V.; S.J.M is a shareholder of and employed by Synvenio B.V.; J.F.A.P.; and T.J.B. are shareholders of Synvenio B.V.; Radboud University and Radboudumc have filed patent applications related to P-SiaFNEtoc (including patent number 11,639,364). MM has filed patents related to Siglec inhibitors (US patent applications 63/421,007 and 63/497,540). All other authors declare that there are no potential competing interests.

## Appendix ASupplementary data

Supplementary Figs. S1–S8 **Fig. S1.** Analysis of ST6GAL1 level in prostate cancer patient tissue. (**a**) Real-time PCR analysis of *ST6GAL1* gene expression levels in 20 prostate cancer tissues from the Walker et al. cohort.^50^ Patients within the ‘metastatic molecular sub-group’ have 6.3-fold upregulated ST6GAL1 compared to the ‘non-metastatic sub-group’ (n = 20, unpaired t test, p = 0.024). (**b**) *ST6GAL1* gene levels are 2.13-fold higher in metastatic CRPC (mCRPC) compared to hormone naïve (HN) disease (n = 20, unpaired t test, p = 0.0023). (**c**) Analysis of ST6GAL1 protein levels in a previously published 96 case TMA (TMA cohort 1). Prostate cancer patients with Gleason grade 7–10 tumours have 2-fold higher ST6GAL1 levels than patients diagnosed with Gleason 6 tumours (unpaired t test, p = 0.0042). There was no significant difference between Gleason 7–8 and Gleason 9–10 tumours in this TMA (unpaired t test, p = 0.4542). Scale bar is 100 μm. **Fig. S2.** Validation of the ST6GAL1 overexpressing luciferase tagged PC3 stable cell line. (**a**,**b**) Real-time PCR analysis of ST6GAL1 gene levels and sandwich ELISA assay analysis of ST6GAL1 protein levels in a panel of prostate cancer cell lines, including LNCaP, CWR22RV1, DU145 and PC3 cells. ST6GAL1 is increased in LNCaP cells treated with 10 nM R1881 (androgens, A+) for 48 h compared to LNCaP cells grown without androgens (steroid deplete, SD). The levels of endogenous ST6GAL1 are lowest in both DU145 and PC3 cells. (**c**) Real time PCR analysis of *ST6GAL1* gene levels in control and ST6GAL1 overexpressing cells, normalised to *GAPDH*. (unpaired t test, p = 0.0131) (**d**) Western blot analysis of ST6GAL1 protein levels in control and ST6GAL1 overexpressing cells. (**e**) Sandwich ELISA assay to detect ST6GAL1 protein levels in cell pellet samples from control and ST6GAL1 overexpressing cells (unpaired t test, p < 0.0001). (**f**) SNA lectin immunofluorescence shows PC3 cells with upregulation of ST6GAL1 have increased levels of α2-6 sialylation (SNA, the lectin from *Sambucus nigra*, recognises α2-6 linked sialylated *N*-glycans) Scale bar is 20 μm. (**g**) SNA lectin flow cytometry also shows PC3 cells with upregulation of ST6GAL1 have significantly increased binding of SNA lectin (p < 0.0001) indicating higher levels of α2,6 sialylated *N*-glycans in cells. (**h**) Analysis of firefly luciferase levels in luciferase tagged PC3 cells shows the control and ST6GAL1 overexpressing cell lines have similar levels of luciferase expression. **Fig. S3.** ST6GAL1 overexpressing PC3 cells cause enhanced bone destruction. Six weeks post tumour cell inoculation via the intra-cardiac route, the mice were euthanised and left tibias were subject to micro-CT analysis. (**a**) Micro-CT analysis shows a 11% reduction of tibial trabecular bone volume (BV/TV) in the mice injected with ST6GAL1 overexpressing PC3 cells (Mann-Whitney test, p = 0.0545). (**b**,**c**) Histomorphometry analysis on tibial endocortical surfaces suggests a 3.6-fold increase in the number of osteoclasts (unpaired t test, p = 0.0268) but a 0.58-fold reduction in the number of osteoblasts (unpaired t test, p = 0.1107). **Fig. S4.** RNA-seq of PC3 prostate cancer cells with upregulation of ST6GAL1 and additional validation. (**a**,**b**) Volcano and MD plots of RNA-seq data from control and ST6GAL1 overexpressing PC3 cells. When ST6GAL1 is overexpressed, bioinformatic analyses identified 776 differentially expressed genes in PC3 cells (adjusted p value < 0.05, Log_2_FC 0.58) (Supplementary Table S2). (**c–e**) Gene Ontology and gene set enrichment analyses of genes regulated by ST6GAL1 revealed PC3 cells overexpressing ST6GAL1 have upregulation in ‘interferon alpha response’, ‘complement system’ and downregulation in ‘epithelial mesenchymal transition’ (EMT) and ‘inflammatory response’. **Fig. S5.** RNA-seq of DU145 prostate cancer cells with upregulation of ST6GAL1 and additional validation. (**a,b**) Volcano and MD plots of RNA-seq data from control and ST6GAL1 overexpressing DU145 cells. When ST6GAL1 is overexpressed, bioinformatic analyses identified 1156 differentially expressed genes in DU145 cells (adjusted p value < 0.05, Log_2_FC 0.58) (Supplementary Table S3).(**c–e**) Gene Ontology and gene set enrichment analyses of genes regulated by ST6GAL1 shows an enrichment in the ‘epithelial mesenchymal transition (EMT)’, ‘inflammatory response’ and ‘IL6 JAK STAT3 signalling’ hallmark signatures. **Fig. S6.** Additional validation of genes and proteins regulated by ST6GAL1 in prostate cancer cells. (**a**) Real-time PCR analysis confirms upregulation of *ID2* in PC3 cells with overexpression of ST6GAL1. Analysis of the TCGA PRAD cohort shows a significant correlation between *ST6GAL1* and *ID2* gene levels (spearman’s correlation 0.18, p value < 0.001). Similarly, analysis of the metastatic CRPC Stand Up to Cancer/Prostate Cancer Foundation (SU2C/PCF) cohort also revealed a significant correlation between *ST6GAL1* and *ID2* gene levels (spearman’s correlation 0.34, p value < 0.001). (**b**) Real-time PCR, western blotting, and immunocytochemistry analysis of DU145 cells confirm downregulation of the tumour suppressor desmoplakin (DSP) in ST6GAL1 overexpressing cells. (**c**) ST6GAL1 regulates secreted IGFBP5 levels in PC3 cells. Sandwich ELISA analysis of IGFBP5 levels in conditioned media samples from PC3 cell overexpressing ST6GAL1. IGFBP5 is significantly increased in PC3 cells overexpressing ST6GAL1 (unpaired t test, p = 0.0003). (**d**,**e**) ST6GAL1 regulates secretion of CSF1 in prostate cancer cells. Sandwich ELISA analysis of CSF1 levels in conditioned media samples from prostate cancer cells with overexpression or knockdown of ST6GAL1. CSF1 is significantly upregulated in DU145 cells with upregulation of ST6GAL1 (unpaired t test, p = 0.003). CSF1 is significantly downregulated in CWR22RV1 cells with shRNA mediated knockdown of ST6GAL1 (unpaired t test, p = 0.008). (**e**) (**f,g**) Treatment of primary murine pre-osteoclasts with conditioned media samples from DU145 cells overexpressing ST6GAL1 significantly increases nuclei number (unpaired t test, p < 0.0001) and size of osteoclasts (unpaired t test, p < 0.0001). **Fig. S7.** Detection of Siglec-2 and Siglec-3 ligands in prostate cancer cells using Siglec-Fc protein immunocytochemistry.^48^ Mutant Siglec-Fc proteins and neuraminidase treatments were included as controls in this study. (**a**) Siglec-Fc binding is abolished in cells treated with neuraminidase (which removes sialic acid from cells). This control was used in all experiments, with representative images for Siglec-3 Fc shown here. Scale bar is 50 μm. (**b,c**) Detection of ligands for Siglec-2 and Siglec-3 in clinical prostate tissue. Siglec-Fc protein immunocytochemistry was used to detect sialoglycans predicted to bind Siglec-2 and Siglec-3 on immune cells. Positive staining was detected in clinical tissue from two prostate cancer patients. No signal was detected when mutant Siglec Fc proteins were used. Scale bar is 50 μm. (**d,e**) High expression of the *Siglec-2* (*CD22*) and *Siglec-3* (CD33) genes in the TCGA PRAD cohorts corelates with reduced disease free progression in patients. (**f**) Validation of ST6GAL1 knockdown in murine RM1 cells. Knockdown of ST6GAL1 in RM1 cells significantly reduced binding of SNA lectin (which binds α2,6 sialylated glycans) detected by SNA lectin immunofluorescence and SNA lectin flow cytometry. Scale bar is 100 μm. **Fig. S8.** Treatment with the sialytransferase inhibitor P-SiaFNEtoc inhibits the sialylation of murine TRAMPC2 and RM1 prostate cancer cells but does not impact cell viability. (**a**) SNA lectin flow cytometry of TRAMPC2 cells treated with 64 μM P-SiaFNEtoc for 72 h (the bar plot and statistical analysis of this data is shown in Fig. 6b). (**b**) Detection of α2,6 linked sialylated glycans in TRAMPC2 cells treated with 64 μM P-SiaFNEtoc for 72 h by immunocytochemistry using SNA lectin (which recognises α2,6 linked sialylated glycans). (**c**) Detection of α2,3 linked sialylated glycans in TRAMPC2 cells treated with 64 μM P-SiaFNEtoc for 72 h by immunocytochemistry using MAL1 lectin (which recognises α2,3 linked sialylated glycans). Scale bar is 20 μm. (**d**) CellTiter-Glo luminescent assays show no change in cell viability for TRAMPC2 cells treated with 64 μM P-SiaFNEtoc for 72 h. (**e**) SNA lectin flow cytometry of RM1 cells treated with 256 μM P-SiaFNEtoc for 72 h (the bar plot and statistical analysis of this data is shown in Fig. 6e). (**f**) Detection of α2,6 linked sialylated glycans in RM1 cells treated with 256 μM P-SiaFNEtoc for 72 h by immunocytochemistry using SNA lectin (which recognises α2-6 linked sialylated glycans). (**g**) Detection of α2,3 linked sialylated glycans in RM1 cells treated with 256 μM P-SiaFNEtoc for 72 h by immunocytochemistry using MAL1 lectin (which recognises α2,3 linked sialylated glycans). Scale bar is 20 μm. (**h**) CellTiter-Glo luminescent assays show no change in cell viability for RM1 cells treated with 256 μM P-SiaFNEtoc for 72 h.

Supplementary Tables S1–S3

Antibody datasheets

Cell line validation

Western blots
