# Supplementary material for: Sialic acid blockade inhibits the metastatic spread of prostate cancer to bone
Source: eBioMedicine. 2024 May 20;104:105163. doi: 10.1016/j.ebiom.2024.105163 (PMC11134892; doi:10.1016/j.ebiom.2024.105163)

Supplementary Figure 1

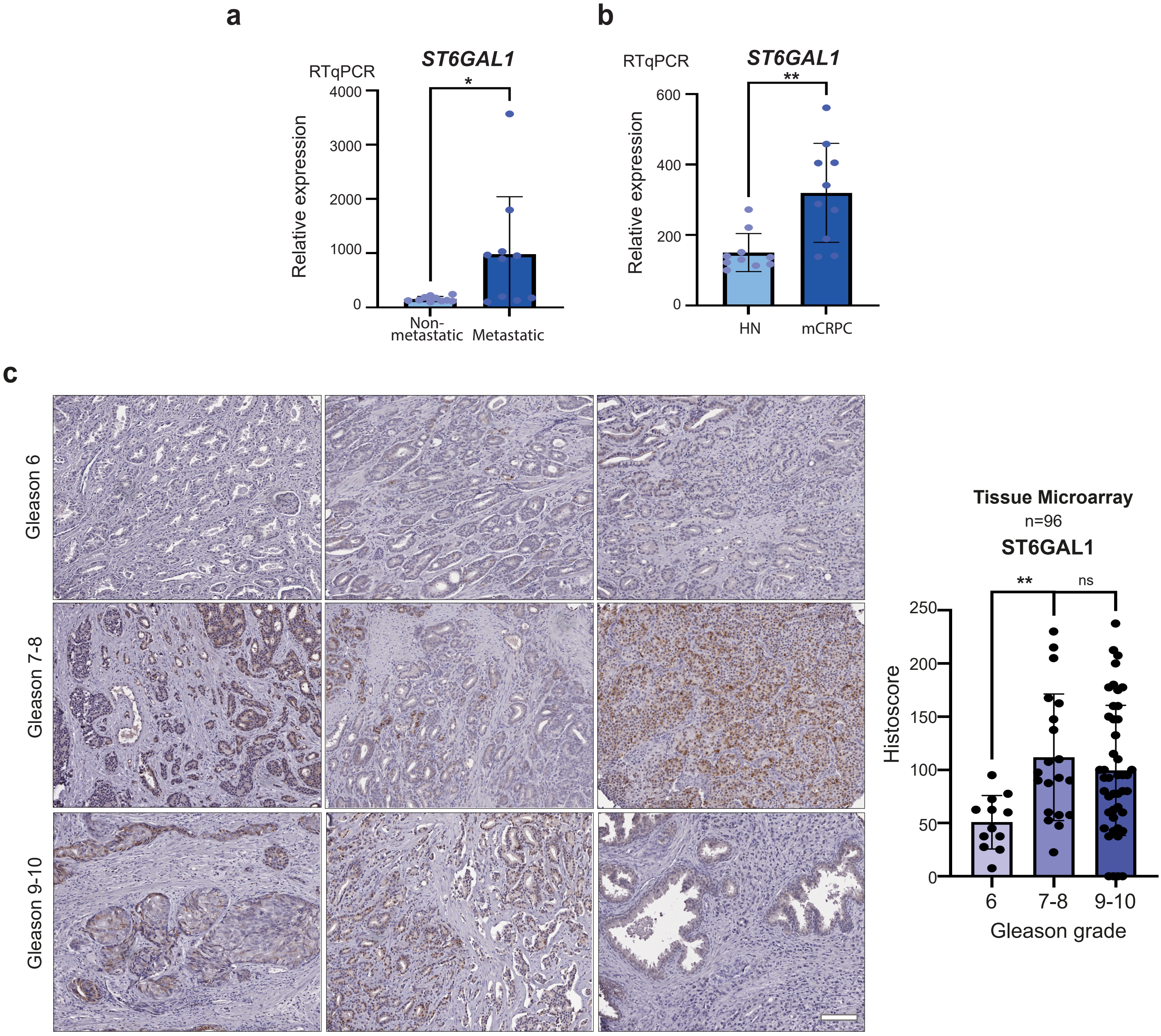

Supplementary Figure 2

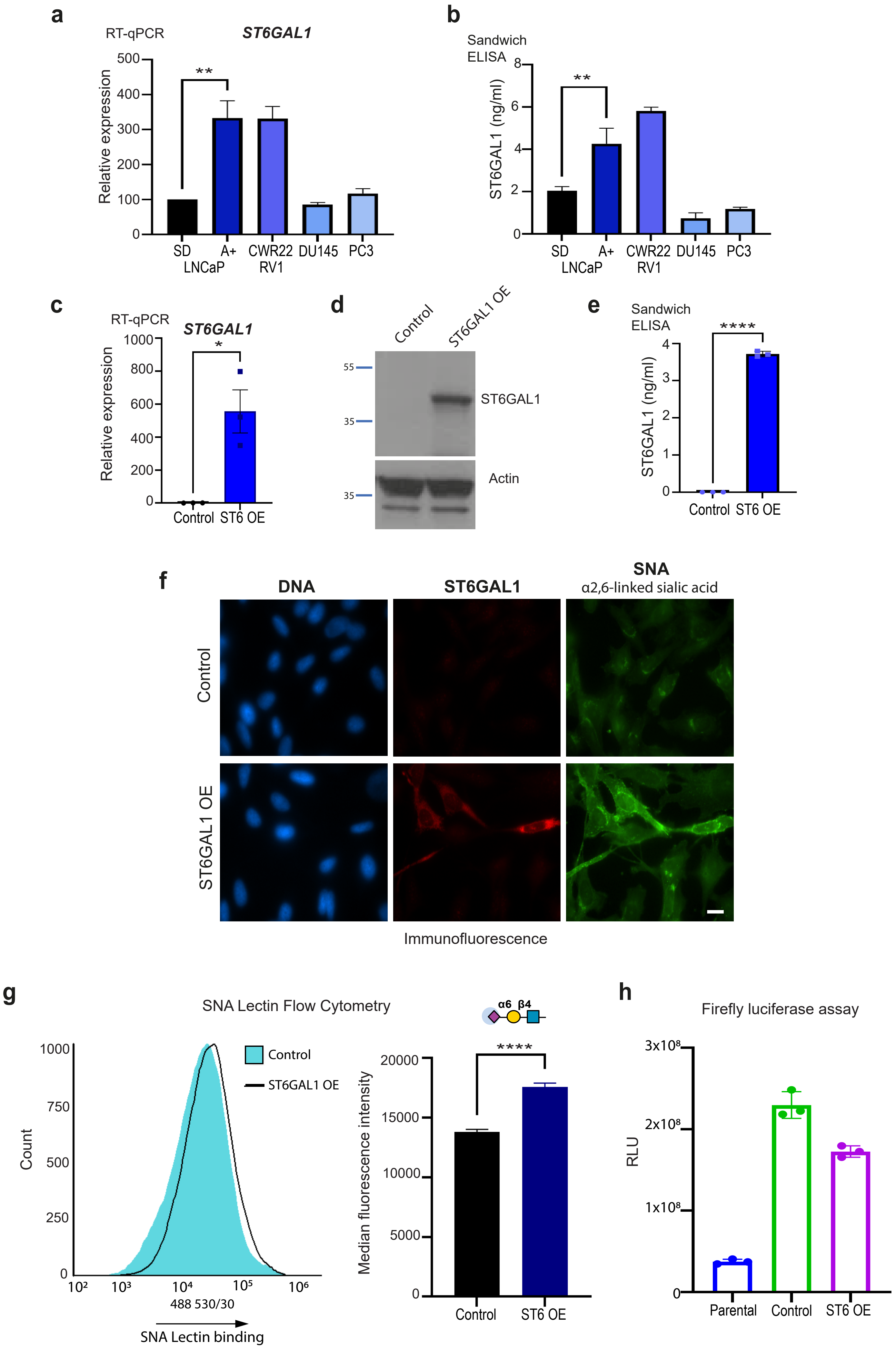

Supplementary Figure 3

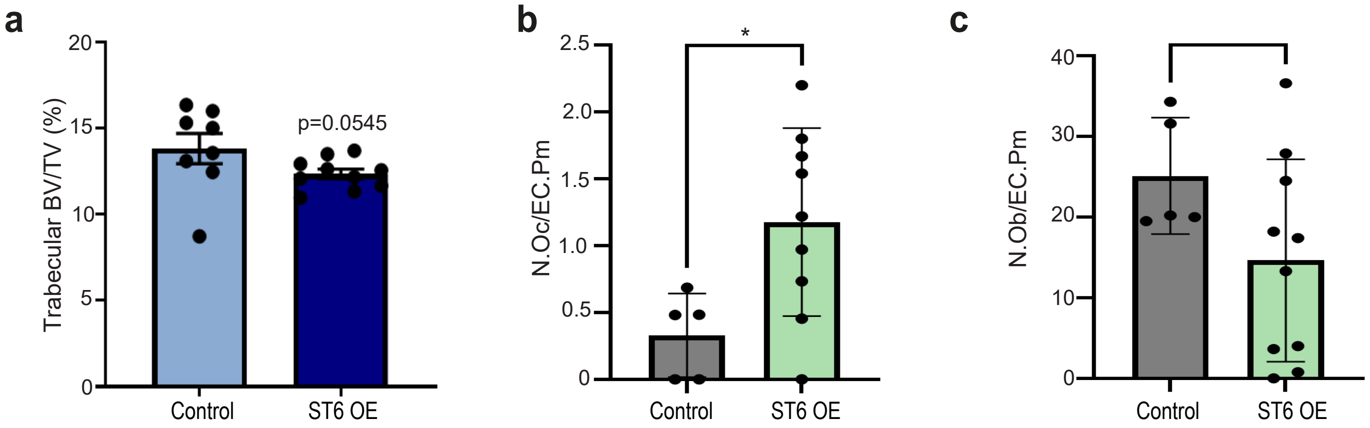

Supplementary Figure 4

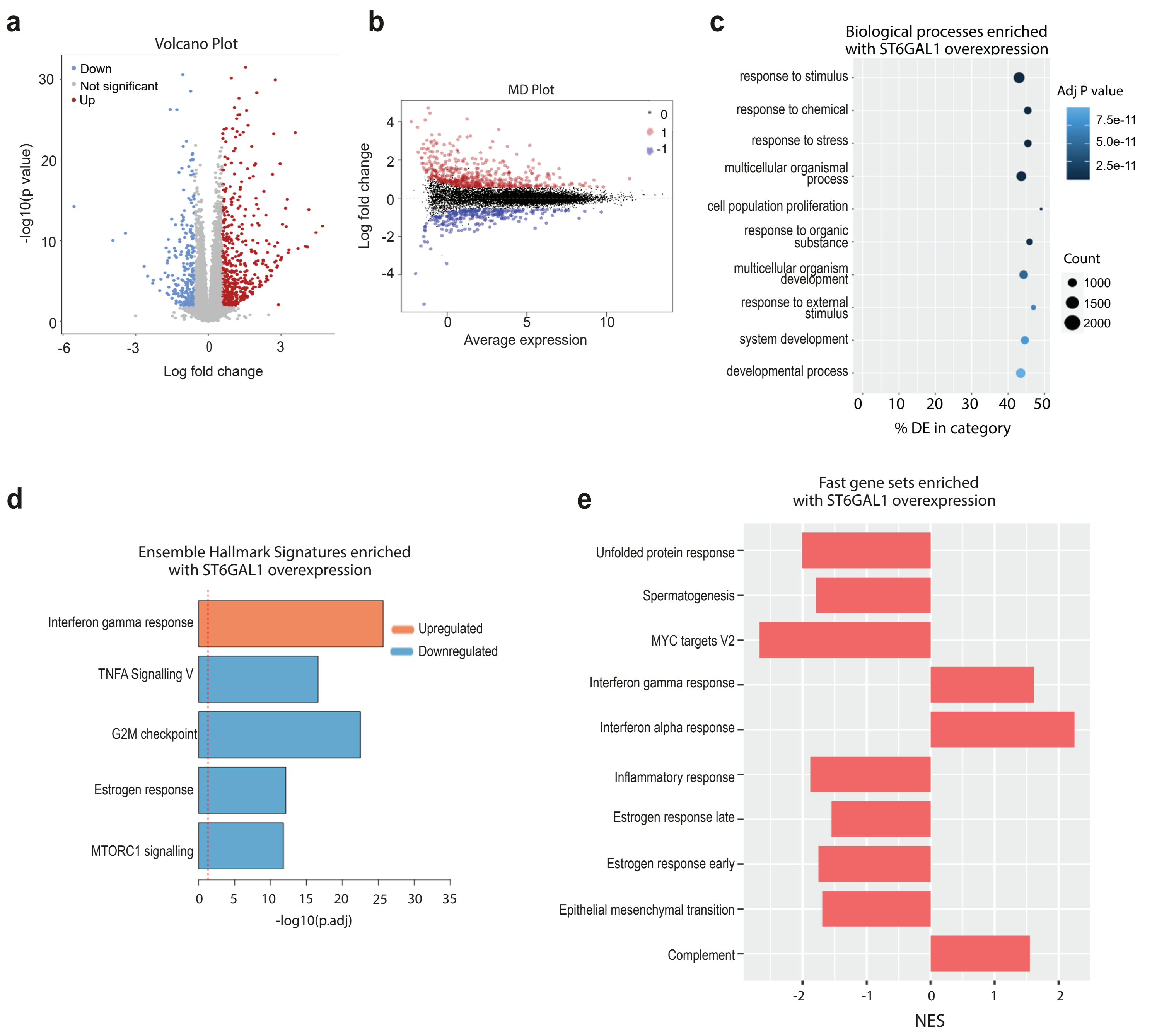

Supplementary Figure 5

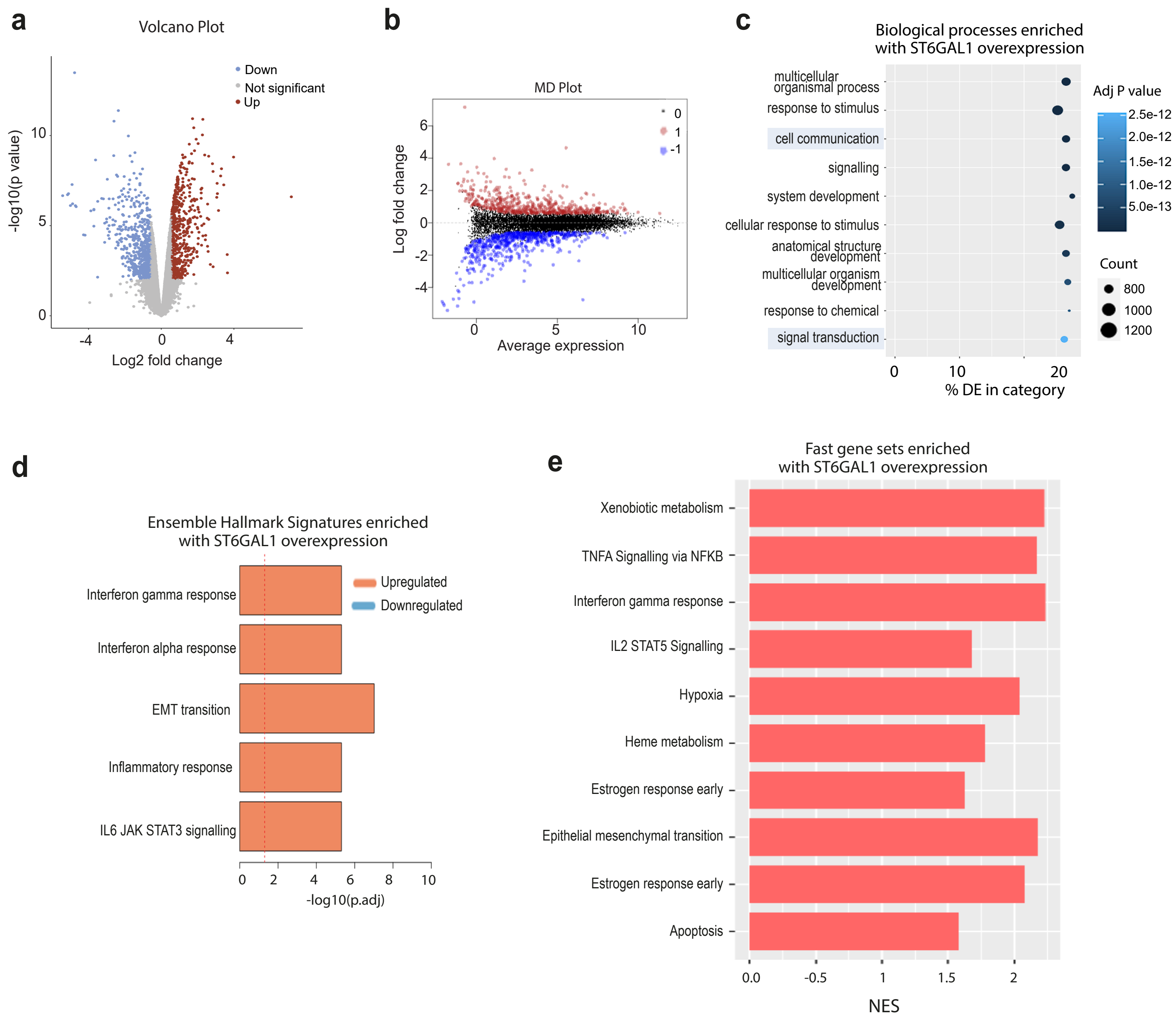

Supplementary Figure 6

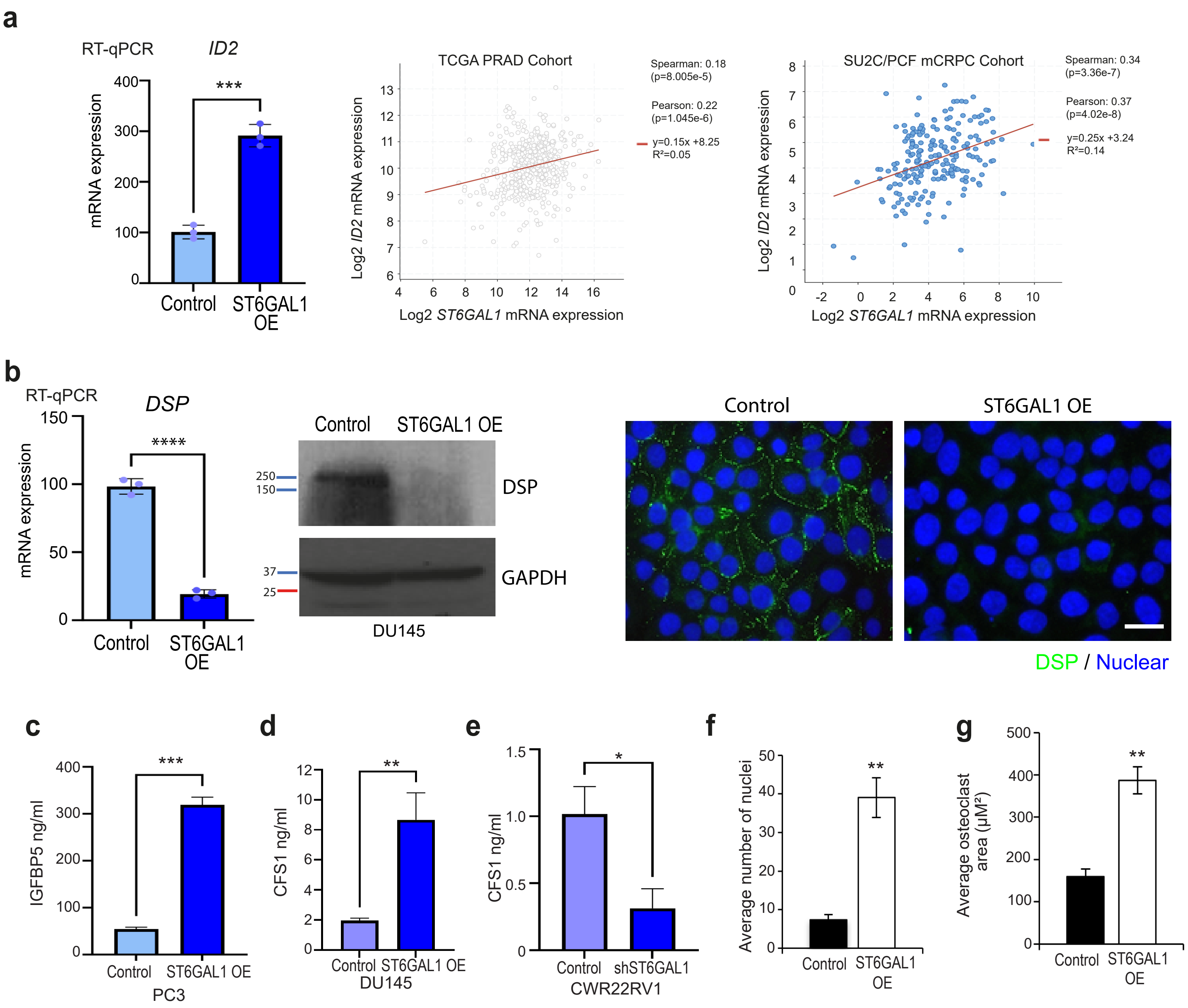

Supplementary Figure 7

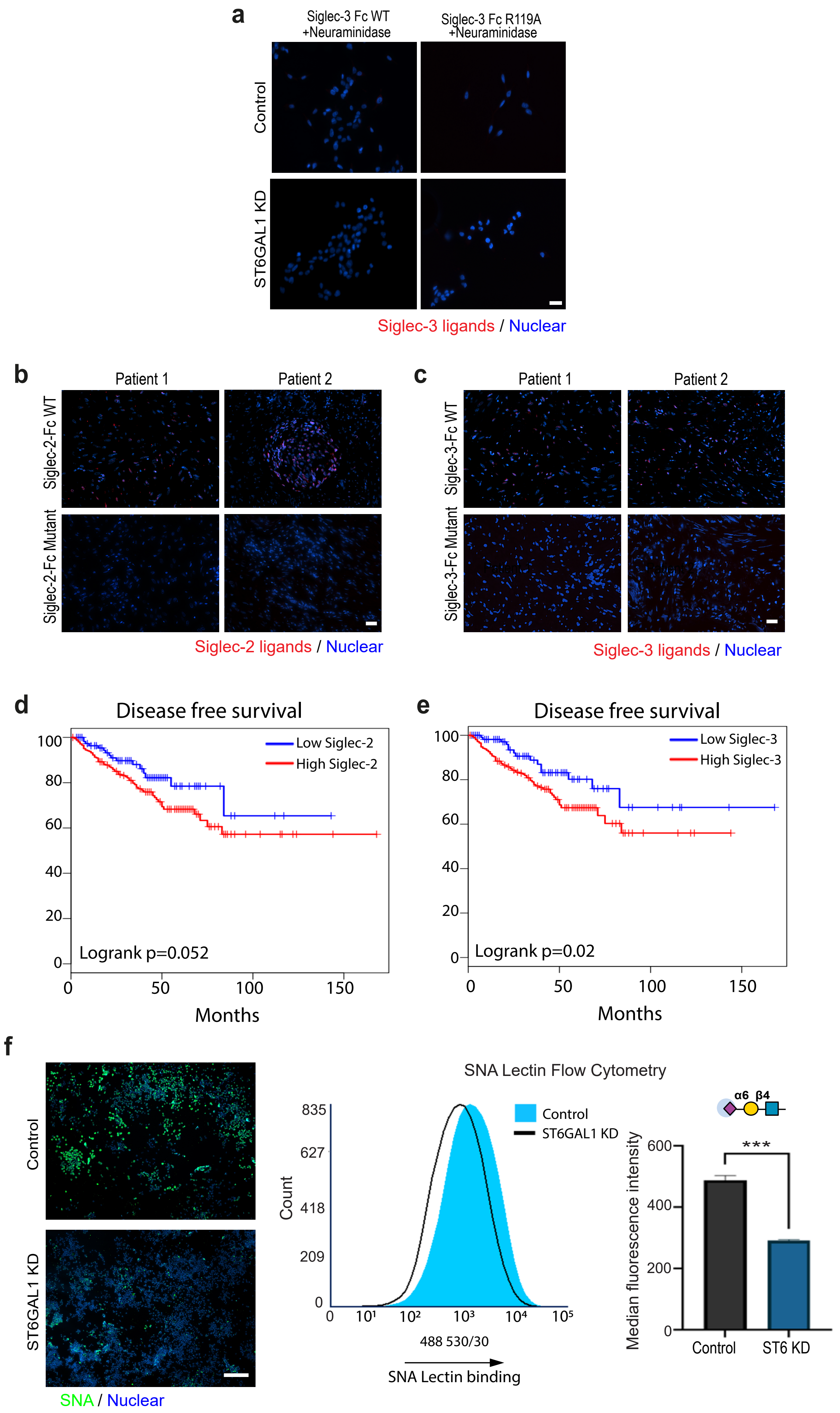

Supplementary Figure 8

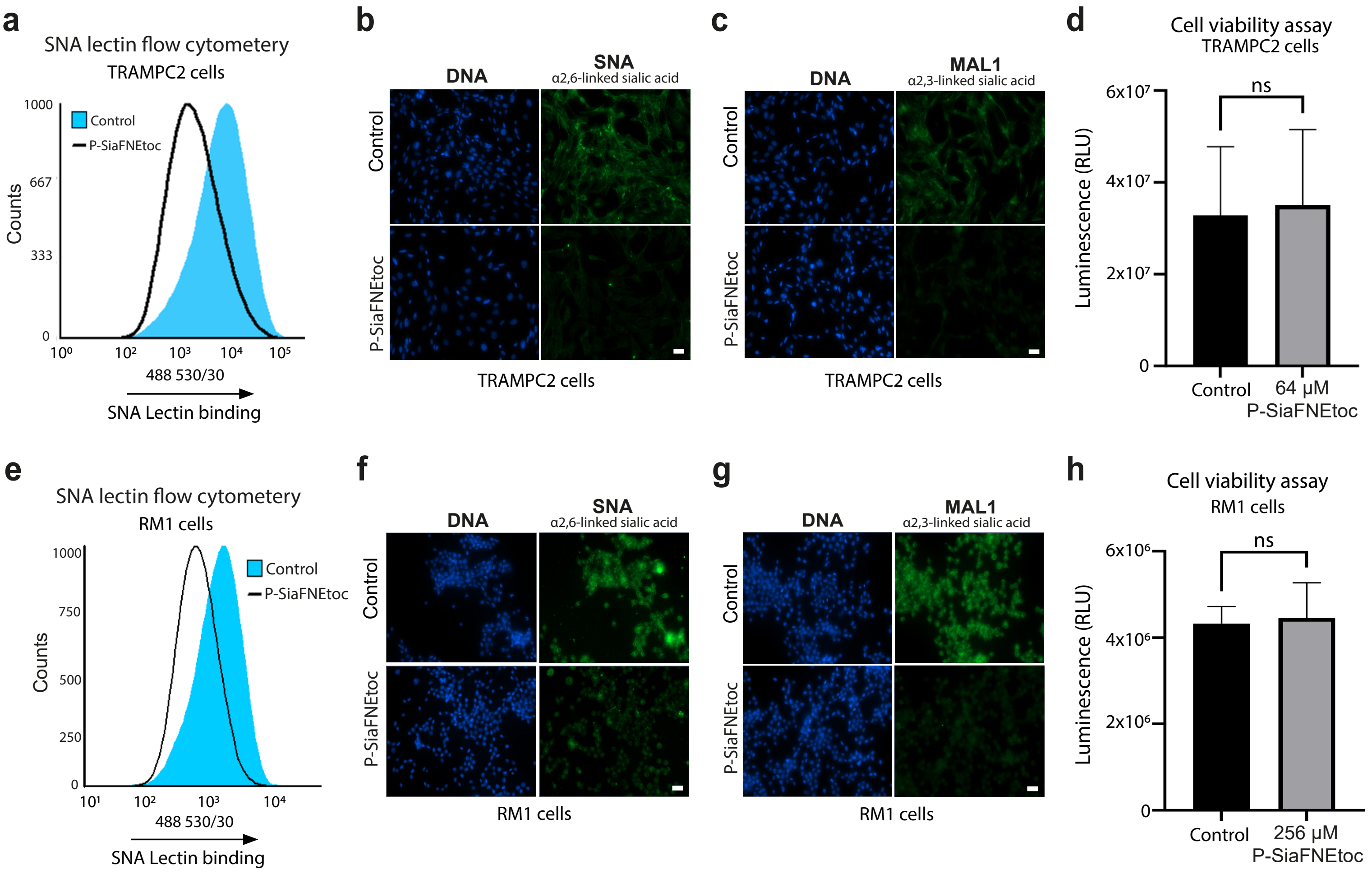

Supplement: Supplementary Figs. S1–S8 — Fig. S1. Analysis of ST6GAL1 level in prostate cancer patient tissue. (a) Real-time PCR analysis of ST6GAL1 gene expression levels in 20 prostate cancer tissues from the Walker et al. cohort.50 Patients within the ‘metastatic molecular sub-group’ have 6.3-fold upregulated ST6GAL1 compared to the ‘non-metastatic sub-group’ (n = 20, unpaired t test, p = 0.024). (b) ST6GAL1 gene levels are 2.13-fold higher in metastatic CRPC (mCRPC) compared to hormone naïve (HN) disease (n = 20, unpaired t test, p = 0.0023). (c) Analysis of ST6GAL1 protein levels in a previously published 96 case TMA (TMA cohort 1). Prostate cancer patients with Gleason grade 7–10 tumours have 2-fold higher ST6GAL1 levels than patients diagnosed with Gleason 6 tumours (unpaired t test, p = 0.0042). There was no significant difference between Gleason 7–8 and Gleason 9–10 tumours in this TMA (unpaired t test, p = 0.4542). Scale bar is 100 μm. Fig. S2. Validation of the ST6GAL1 overexpressing luciferase tagged PC3 stable cell line. (a,b) Real-time PCR analysis of ST6GAL1 gene levels and sandwich ELISA assay analysis of ST6GAL1 protein levels in a panel of prostate cancer cell lines, including LNCaP, CWR22RV1, DU145 and PC3 cells. ST6GAL1 is increased in LNCaP cells treated with 10 nM R1881 (androgens, A+) for 48 h compared to LNCaP cells grown without androgens (steroid deplete, SD). The levels of endogenous ST6GAL1 are lowest in both DU145 and PC3 cells. (c) Real time PCR analysis of ST6GAL1 gene levels in control and ST6GAL1 overexpressing cells, normalised to GAPDH. (unpaired t test, p = 0.0131) (d) Western blot analysis of ST6GAL1 protein levels in control and ST6GAL1 overexpressing cells. (e) Sandwich ELISA assay to detect ST6GAL1 protein levels in cell pellet samples from control and ST6GAL1 overexpressing cells (unpaired t test, p < 0.0001). (f) SNA lectin immunofluorescence shows PC3 cells with upregulation of ST6GAL1 have increased levels of α2-6 sialylation (SNA, the lectin from Sambucus nigra, [file mmc1.pdf]
