# Supplementary material for: Sialic acid blockade inhibits the metastatic spread of prostate cancer to bone
Source: eBioMedicine. 2024 May 20;104:105163. doi: 10.1016/j.ebiom.2024.105163 (PMC11134892; doi:10.1016/j.ebiom.2024.105163)
Supplement: Antibody datasheets [file mmc3.pdf]

10320 Camino Santa Fe, Suite G
San Diego, CA 92121
Tel: 858.875.1900 Fax: 858.875.1999

ST6GAL1 Antibody (Center)
Affinity Purified Rabbit Polyclonal Antibody (Pab)
Catalog # AP19891c

Specification

ST6GAL1 Antibody (Center) - Product Information

Application	WB,E
Primary Accession	P15907
Other Accession	NP_775324.1
Reactivity	Human
Host	Rabbit
Clonality	Polyclonal
Isotype	Rabbit IgG
Calculated MW	46605
Antigen Region	178-206

ST6GAL1 Antibody (Center) - Additional Information

Gene ID 6480

Other Names
Beta-galactoside alpha-2, 6-sialyltransferase 1, Alpha 2, 6-ST 1, B-cell antigen CD75, CMP-N-acetylneuraminate-beta-galactosamide-alpha-2, 6-sialyltransferase 1, ST6Gal I, ST6GalI, Sialyltransferase 1, ST6GAL1, SIAT1

Target/Specificity
This ST6GAL1 antibody is generated from rabbits immunized with a KLH conjugated synthetic peptide between 178-206 amino acids from the Central region of human ST6GAL1.

Dilution
WB~~1:1000

Format
Purified polyclonal antibody supplied in PBS with 0.09% (W/V) sodium azide. This antibody is purified through a protein A column, followed by peptide affinity purification.

Storage
Maintain refrigerated at 2-8°C for up to 2 weeks. For long term storage store at -20°C in small aliquots to prevent freeze-thaw cycles.

Precautions
ST6GAL1 Antibody (Center) is for research use only and not for use in diagnostic or therapeutic procedures.

ST6GAL1 Antibody (Center) - Protein Information

Name ST6GAL1

Synonyms SIAT1

Function Transfers sialic acid from CMP-sialic acid to galactose- containing acceptor substrates.

Cellular Location
Golgi apparatus, Golgi stack membrane; Single-pass type II membrane protein. Secreted. Note=Membrane-bound form in trans cisternae of Golgi. Secreted into the body fluid

ST6GAL1 Antibody (Center) - Protocols

Provided below are standard protocols that you may find useful for product applications.

- Western Blot
- Blocking Peptides
- Dot Blot
- Immunohistochemistry
- Immunofluorescence
- Immunoprecipitation
- Flow Cytomety
- Cell Culture

ST6GAL1 Antibody (Center) - Images

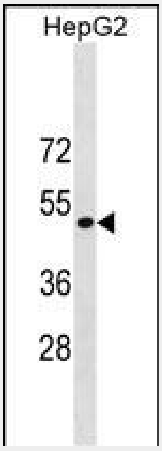

ST6GAL1 Antibody (Center) (Cat. #AP19891c) western blot analysis in HepG2 cell line lysates (35ug/lane).This demonstrates the ST6GAL1 antibody detected the ST6GAL1 protein (arrow).

ST6GAL1 Antibody (Center) - Background

This gene encodes a member of glycosyltransferase family 29. The encoded protein is a type II membrane protein that catalyzes the transfer of sialic acid from CMP-sialic acid to galactose-containing substrates. The protein, which is normally found in the Golgi but can be proteolytically processed to a soluble form, is involved in the generation of the cell-surface carbohydrate determinants and differentiation antigens HB-6, CD75, and CD76. This gene has been incorrectly referred to as CD75. Three transcript variants encoding two different isoforms have been described.

ST6GAL1 Antibody (Center) - References

Rose, J.E., et al. Mol. Med. 16 (7-8), 247-253 (2010) :
Mondal, S., et al. Leuk. Res. 34(4):463-470(2010)
Lee, M., et al. Oncol. Rep. 23(3):757-761(2010)
Daly, A.K., et al. Nat. Genet. 41(7):816-819(2009)
Costa-Nogueira, C., et al. BMC Cancer 9, 431 (2009) :

Desmoplakin Antibody

Rabbit Polyclonal

Antigen Affinity Purified		Protein ID	NP_004406.2
Catalog No.	A303-355A	GeneID	1832
Lot No.	A303-355A-1		

**APPLICATIONS**	WB, IP
**SPECIES REACTIVITY**	Human
**AMOUNT**	100 µl
**CONCENTRATION**	1000 µg/ml
**STORAGE/SHELF LIFE**	2 - 8° C / 1 year from date of receipt
**PHYSICAL STATE**	Liquid
**BUFFER**	Tris-citrate/phosphate buffer, pH 7 to 8 containing 0.09% Sodium Azide
**ISOTYPE**	IgG
**ORIGIN**	USA

PRODUCTION PROCEDURES

Antibody was affinity purified using an epitope specific to Desmoplakin immobilized on solid support.

The epitope recognized by A303-355A maps to a region between residue 1600 and 1650 of human Desmoplakin using the numbering given in entry NP_004406.2 (GeneID 1832).

Antibody concentration was determined by extinction coefficient: absorbance at 280 nm of 1.4 equals 1.0 mg of IgG.

APPLICATIONS

Centrifuge tube to remove product from lid. Optimal working dilutions should be determined experimentally by the investigator. Prepare working dilution immediately before use.

Western Blot	1:2,000 - 1:10,000
Immunoprecipitation	2 - 10 µg/mg lysate

APPLICATION NOTES

Western blot of immunoprecipitates performed using Normal Pig Serum (Cat. No. S100-020), Goat anti-Rabbit Light Chain HRP Conjugate (Cat. No. A120-113P) and 3-8% SDS-PAGE (link to IP-western blot protocol in Additional Info section below).

Western blot of lysates performed using standard western blot reagents and 3-8% SDS-PAGE.

ADDITIONAL INFO

https://www.bethyl.com/product/A303-355A

Use the link above to view SDS, a current list of citations, and other product specific information.

IP-western blot protocol: https://www.bethyl.com/content/protocol_IP_WB

This document certifies that this product has met all of the quality control standards defined by Bethyl Laboratories, Inc.

Eric McIntush, PhD | Chief Scientific Officer — Date: June 21, 2019

For in vitro laboratory use only. Not for any clinical, therapeutic or diagnostic use in humans or animals. Not for human or animal consumption. This product may not be resold or modified for resale without the prior written approval of Bethyl Laboratories, Inc. The information provided in this data sheet is believed to be correct but does not purport to be all-inclusive and is intended to be used as a guide. Bethyl Laboratories, Inc. shall not be liable or responsible in any way for use of either this information or the material supplied. Disposal of hazardous material may be subject to federal, state or local laws or regulations.

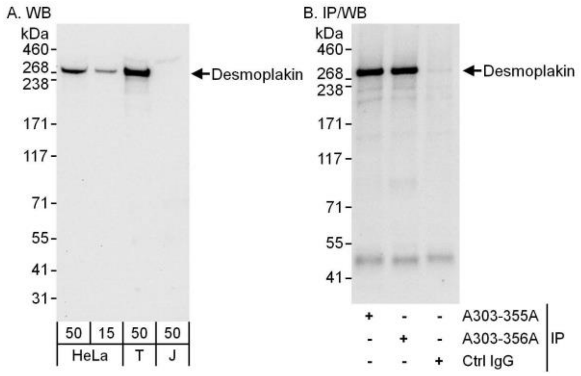

Detection of human Desmoplakin by western blot and immunoprecipitation. *Samples:* Whole cell lysate from HeLa (15 and 50 µg for WB; 1 mg for IP, 20% of IP loaded), HEK293T (T; 50 µg), and Jurkat (J; 50 µg) cells. *Antibodies:* Affinity purified rabbit anti-Desmoplakin antibody A303-355A used for WB at 0.1 µg/ml (A) and 1 µg/ml (B) and used for IP at 6 µg/mg lysate. Desmoplakin was also immunoprecipitated by rabbit anti-Desmoplakin antibody A303-356A, which recognizes a downstream epitope. *Detection:* Chemiluminescence with exposure times of 10 seconds (A and B).

For in vitro laboratory use only. Not for any clinical, therapeutic or diagnostic use in humans or animals. Not for human or animal consumption. This product may not be resold or modified for resale without the prior written approval of Bethyl Laboratories, Inc. The information provided in this data sheet is believed to be correct but does not purport to be all-inclusive and is intended to be used as a guide. Bethyl Laboratories, Inc. shall not be liable or responsible in any way for use of either this information or the material supplied. Disposal of hazardous material may be subject to federal, state or local laws or regulations.

A2066 Sigma-Aldrich

Anti-Actin antibody produced in rabbit

Synonym(s):
Actin Antibody Sigma, Anti Actin, Anti Actin Antibody Western Blot, Anti Actin Antibody for western blot - Anti-Actin antibody produced in rabbit, Anti Actin Sigma, Sigma Actin Antibody

PROPERTIES

biological source	rabbit
Quality Level	200
conjugate	unconjugated
antibody form	affinity isolated antibody
antibody product type	primary antibodies
clone	polyclonal
form	buffered aqueous solution
mol wt	antigen 42 kDa
species reactivity	wide range, vertebrates, human, slime mold, amoeba, chicken
enhanced validation	independent<br>Learn more about **Antibody Enhanced Validation**

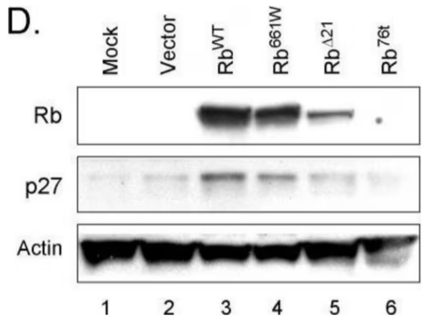

Western Blotting
JOURNAL CITATION: *Rb induces a proliferative arrest and curtails Brn-2 expression in retinoblastoma cells.* By: Cobrinik, D., Francis, R. O., et al. in Mol Cancer, 2006. PubMed ID: 17163992
Image collected and cropped by CiteAb from the following publication, (http://molecular-cancer.biomedcentral.com/articles/10.1186/1476-4598-5-72), provided under a CC-BY license. Image might reflect a new usage that is not reflected in claims on product description or independently verified by Merck KGaA, Darmstadt, Germany. For Research Use Only. Not for use in diagnostic procedures.

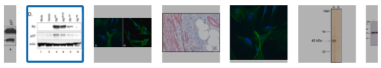

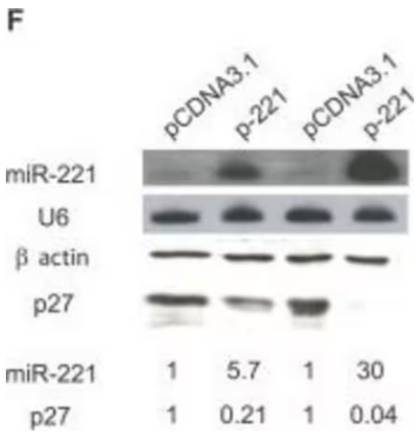

Western Blotting
JOURNAL CITATION: *The inhibition of the highly expressed miR-221 and miR-222 impairs the growth of prostate carcinoma xenografts in mice.* By: Mercatelli, N., Coppola, V., et al. in PLoS One, 2008. PubMed ID: 19107213
Image collected and cropped by CiteAb from the following publication, (http://dx.plos.org/10.1371/journal.pone.0004029), provided under a CC-BY license. Image might reflect a new usage that is not reflected in claims on product description or independently verified by Merck KGaA, Darmstadt, Germany. For Research Use Only. Not for use in diagnostic procedures.

10320 Camino Santa Fe, Suite G
San Diego, CA 92121
Tel: 858.875.1900 Fax: 858.875.1999

GAPDH Antibody (C-term R248)

Purified Rabbit Polyclonal Antibody (Pab)

Catalog # AP7873b

Specification

GAPDH Antibody (C-term R248) - Product Information

Application	**IF, WB, IHC-P, FC,E**
Primary Accession	P04406
Other Accession	P04797, P00355, P16858, P00356
Reactivity	**Human**
Predicted	**Chicken, Mouse, Pig, Rat**
Host	**Rabbit**
Clonality	**Polyclonal**
Isotype	**Rabbit IgG**
Calculated MW	**36053**
Antigen Region	**233-259**

GAPDH Antibody (C-term R248) - Additional Information

Gene ID 2597

Other Names
Glyceraldehyde-3-phosphate dehydrogenase, GAPDH, Peptidyl-cysteine S-nitrosylase GAPDH, 2699-, GAPDH, GAPD

Target/Specificity
This GAPDH antibody is generated from rabbits immunized with a KLH conjugated synthetic peptide between 233-259 amino acids from the C-terminal region of human GAPDH.

Dilution
IF~~1:10~50
WB~~1:1000
IHC-P~~1:50~100
FC~~1:10~50

Format
Purified polyclonal antibody supplied in PBS with 0.09% (W/V) sodium azide. This antibody is prepared by Saturated Ammonium Sulfate (SAS) precipitation followed by dialysis against PBS.

Storage
Maintain refrigerated at 2-8°C for up to 2 weeks. For long term storage store at -20°C in small aliquots to prevent freeze-thaw cycles.

Precautions
GAPDH Antibody (C-term R248) is for research use only and not for use in diagnostic or therapeutic procedures.

GAPDH Antibody (C-term R248) - Protein Information

Name GAPDH {ECO:0000303|PubMed:2987855, ECO:0000312|HGNC:HGNC:4141}

Function Has both glyceraldehyde-3-phosphate dehydrogenase and nitrosylase activities, thereby playing a role in glycolysis and nuclear functions, respectively (PubMed:3170585, PubMed:11724794). Glyceraldehyde-3-phosphate dehydrogenase is a key enzyme in glycolysis that catalyzes the first step of the pathway by converting D- glyceraldehyde 3-phosphate (G3P) into 3-phospho-D-glyceroyl phosphate (PubMed:3170585, PubMed:11724794). Modulates the organization and assembly of the cytoskeleton (By similarity). Facilitates the CHP1- dependent microtubule and membrane associations through its ability to stimulate the binding of CHP1 to microtubules (By similarity). Component of the GAIT (gamma interferon-activated inhibitor of translation) complex which mediates interferon-gamma-induced transcript-selective translation inhibition in inflammation processes (PubMed:23071094). Upon interferon-gamma treatment assembles into the GAIT complex which binds to stem loop-containing GAIT elements in the 3'-UTR of diverse inflammatory mRNAs (such as ceruplasmin) and suppresses their translation (PubMed:23071094). Also plays a role in innate immunity by promoting TNF-induced NF-kappa-B activation and type I interferon production, via interaction with TRAF2 and TRAF3, respectively (PubMed:23332158, PubMed:27387501). Participates in nuclear events including transcription, RNA transport, DNA replication and apoptosis (By similarity). Nuclear functions are probably due to the nitrosylase activity that mediates cysteine S-nitrosylation of nuclear target proteins such as SIRT1, HDAC2 and PRKDC (By similarity).

Cellular Location
Cytoplasm, cytosol. Nucleus {ECO:0000250|UniProtKB:P04797}. Cytoplasm, perinuclear region. Membrane Cytoplasm, cytoskeleton {ECO:0000250|UniProtKB:P04797} Note=Translocates to the nucleus following S-nitrosylation and interaction with SIAH1, which contains a nuclear localization signal (By similarity). Postnuclear and Perinuclear regions (PubMed:12829261) {ECO:0000250|UniProtKB:P04797, ECO:0000269|PubMed:12829261}

GAPDH Antibody (C-term R248) - Protocols

Provided below are standard protocols that you may find useful for product applications.

- Western Blot
- Blocking Peptides
- Dot Blot
- Immunohistochemistry
- Immunofluorescence
- Immunoprecipitation
- Flow Cytomety
- Cell Culture

GAPDH Antibody (C-term R248) - Images

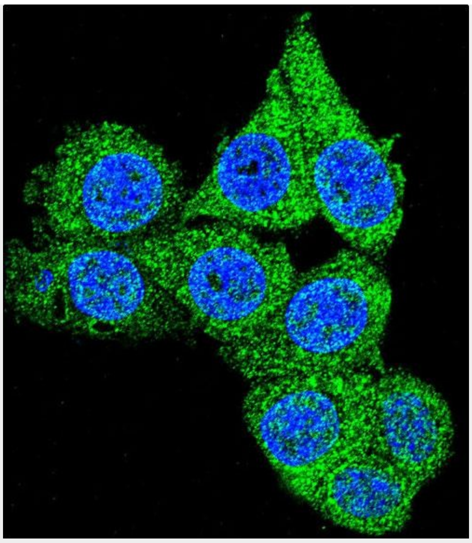

Confocal immunofluorescent analysis of GAPDH Antibody (C-term R248)(Cat#AP7873b) with Hela cell followed by Alexa Fluor 488-conjugated goat anti-rabbit IgG (green). DAPI was used to stain the cell nuclear (blue).

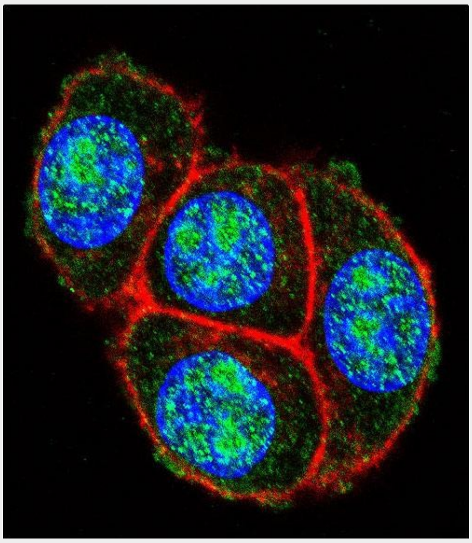

Confocal immunofluorescent analysis of GAPDH Antibody (C-term R248)(Cat#AP7873b) with Hela cell followed by Alexa Fluor 488-conjugated goat anti-rabbit IgG (green). Actin filaments have been labeled with Alexa Fluor 555 phalloidin (red).DAPI was used to stain the cell nuclear (blue).

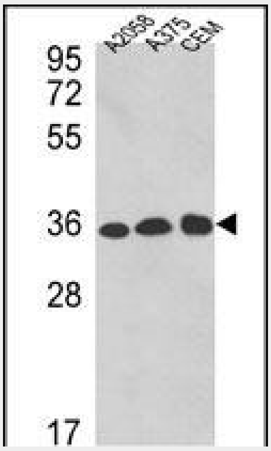

10320 Camino Santa Fe, Suite G
San Diego, CA 92121
Tel: 858.875.1900 Fax: 858.875.1999

Western blot analysis of GAPDH Antibody (C-term R248) (Cat.#AP7873b) in A2058, A375, CEM cell line lysates (35ug/lane). GAPDH (arrow) was detected using the purified Pab.

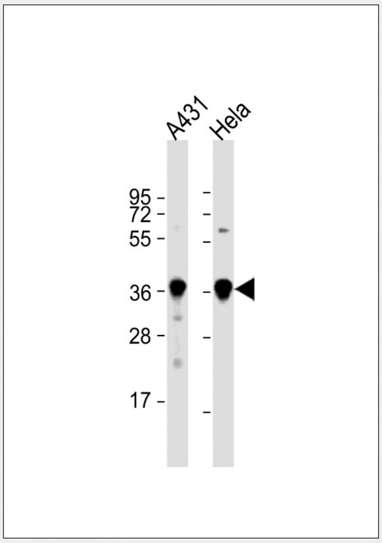

All lanes : Anti-GAPDH Antibody (C-term R248) at 1:1000 dilution Lane 1: A431 whole cell lysate Lane 2: Hela whole cell lysate Lysates/proteins at 20 µg per lane. Secondary Goat Anti-Rabbit IgG, (H+L), Peroxidase conjugated at 1/10000 dilution. Predicted band size : 36 kDa Blocking/Dilution buffer: 5% NFDM/TBST.

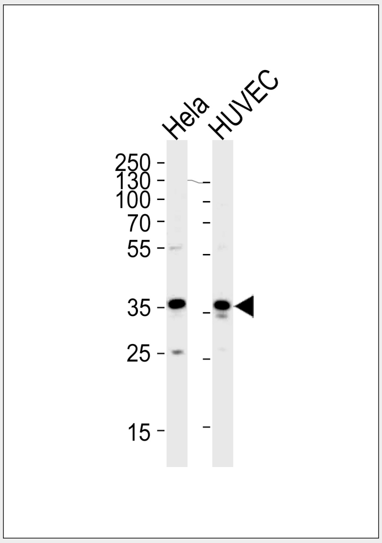

Western blot analysis of lysates from Hela,HUVEC cell line (from left to right),using GAPDH Antibody (C-term R248)(Cat. #AP7873b).AP7873b was diluted at 1:1000 at each lane. A goat anti-rabbit IgG H&L(HRP) at 1:5000 dilution was used as the secondary antibody.Lysates at 35ug per lane.

abcepta
10320 Camino Santa Fe, Suite G
San Diego, CA 92121
Tel: 858.875.1900 Fax: 858.875.1999

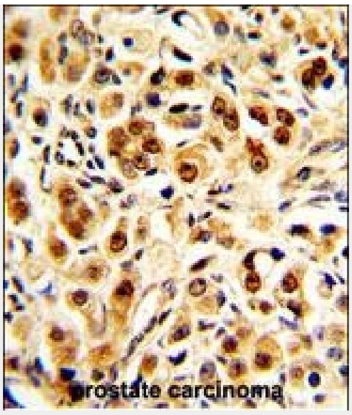

Formalin-fixed and paraffin-embedded human prostate carcinoma with GAPDH Antibody (C-term R248), which was peroxidase-conjugated to the secondary antibody, followed by DAB staining. This data demonstrates the use of this antibody for immunohistochemistry; clinical relevance has not been evaluated.

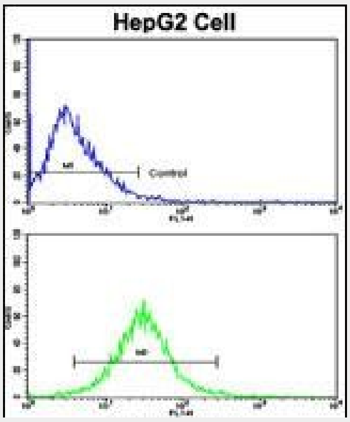

Flow cytometric analysis of HepG2 cells using GAPDH Antibody (C-term R248)(bottom histogram) compared to a negative control cell (top histogram). FITC-conjugated goat-anti-rabbit secondary antibodies were used for the analysis.

GAPDH Antibody (C-term R248) - Background

GAPDH catalyzes an important energy-yielding step in carbohydrate metabolism, the reversible oxidative phosphorylation of glyceraldehyde-3-phosphate in the presence of inorganic phosphate and nicotinamide adenine dinucleotide (NAD). The enzyme exists as a tetramer of identical chains.

GAPDH Antibody (C-term R248) - References

Azam,S., J. Biol. Chem. 283 (45), 30632-30641 (2008)
Lu,J., Biosci. Biotechnol. Biochem. 72 (9), 2432-2435 (2008)
Zhou,Y., Mol. Cancer Res. 6 (8), 1375-1384 (2008)

GAPDH Antibody (C-term R248) - Citations

- An ancient germ cell-specific RNA-binding protein protects the germline from cryptic splice site poisoning.
- Effects of secreted frizzled-related protein 1 on proliferation, migration, invasion, and apoptosis of colorectal cancer cells.
- Metalloproteases meprin-α (MEP1A) is a prognostic biomarker and promotes proliferation and invasion of colorectal cancer.
