# Supplementary material for: Sialic acid blockade inhibits the metastatic spread of prostate cancer to bone
Source: eBioMedicine. 2024 May 20;104:105163. doi: 10.1016/j.ebiom.2024.105163 (PMC11134892; doi:10.1016/j.ebiom.2024.105163)
Supplement: Cell line validation [file mmc4.pdf]

Cell Line Authentication Report for Case Number C-30186-1

We have now completed DNA analysis of the samples presented for Cell Line Authentication

A DNA Profile has been generated from the sample provided by Newcastle University and compared to the relevant profile located on the Cellosaurus Database. A Matching Percentage has been calculated and a value of 97% obtained. Please refer to the detailed results on the second page for further information and interpretation.

Analysis has been conducted using 17 loci allowing the probability of identity from identical matches an approximate power of discrimination of $1x10^{14}$ (1 in 100 trillion). Please note that some cell lines may exhibit genetic instability as they proliferate, leading to discrepancies within the DNA profiles examined.

Report Date 22/11/2023

This report shall not be reproduced, except in full, without written approval of the laboratory. The validity of these results depends on the quality of the sample provided and the identification of samples being correct.

northgene™
A BIOFORTUNA brand

UKAS TESTING 7816

Laboratory Report

Test Requested	Cell Line Authentication
Case Number	C-30186-1
Date Sample Received	13/11/2023
Date Sample Tested	13/11/2023
Date Sample Reported	22/11/2023

Sample Name / Cell Line	Cell Line Source / Profile Source	Sample Number	DNA Number
CWR22RV1	Newcastle University	S-1068834	D-1068834
22Rv1 (CVCL_1045)	Cellosaurus Database	N/A	N/A

Table of Allelic Data

	Genotypes		
STR Locus	CWR22RV1 (Test Sample)	22Rv1 (CVCL_1045) (Comparison Sample)	Match vs. Mis-Match
D5S818	11 | 13	11 | 12 | 13	Mis-Match
D13S317	9 | 12	9 | 12	Match
D7S820	9 | 10 | 11	9 | 10 | 11	Match
D16S539	12 | 12	12 | 12	Match
vWA	15 | 21	15 | 21	Match
TH01	6 | 9.3	6 | 9.3	Match
TPOX	8 | 8	8 | 8	Match
CSF1PO	10 | 11	10 | 11	Match
AMEL	X | Y	X | Y	Match
D3S1358	15 | 15	15 | 15	Match
D21S11	30 | 30	30 | 30	Match
D18S51	13 | 14	13 | 14	Match
Penta E	5 | 13	5 | 13	Match
Penta D	9 | 12	9 | 12	Match
D8S1179	13 | 14	12 | 13 | 14	Mis-Match
FGA	20 | 23	20 | 23	Match
D19S433	13 | 14	13 | 14	Match
D2S1338	17 | 18	17 | 18	Match

Matching Percentage: **97%**
Outcome: **Related**

The outcome percentage is calculated using the Tanabe algorithm. The outcome is designated one of the following statements based upon the outcome percentage:

Related (>80% match)	The Cell Lines are considered to be related.
Inconclusive (60-79% match)	Further profiling is required to determine whether the profiles are related.
No Match (59%>)	It is considered that the two cell lines are unrelated.

Reported By Ms. Ish Jones (Laboratory Scientist)

Authorised By Ms. Eleanor Ralston (Senior Scientist)

These test results should only be used in conjunction with a client's information. The results relate only to the items sampled, as received and tested at this time. DNA analysis as tested by PowerPlex® Fusion.

Cell Line Authentication Report for Case Number C-30186-3

We have now completed DNA analysis of the samples presented for Cell Line Authentication

A DNA Profile has been generated from the sample provided by Newcastle University and compared to the relevant profile located on the Cellosaurus Database. A Matching Percentage has been calculated and a value of 95% obtained. Please refer to the detailed results on the second page for further information and interpretation.

Analysis has been conducted using 17 loci allowing the probability of identity from identical matches an approximate power of discrimination of $1x10^{14}$ (1 in 100 trillion). Please note that some cell lines may exhibit genetic instability as they proliferate, leading to discrepancies within the DNA profiles examined.

Report Date 22/11/2023

This report shall not be reproduced, except in full, without written approval of the laboratory. The validity of these results depends on the quality of the sample provided and the identification of samples being correct.

northgene™
A BIOFORTUNA brand

UKAS TESTING 7816

Laboratory Report

Test Requested	Cell Line Authentication
Case Number	C-30186-3
Date Sample Received	13/11/2023
Date Sample Tested	13/11/2023
Date Sample Reported	22/11/2023

Sample Name / Cell Line	Cell Line Source / Profile Source	Sample Number	DNA Number
DU145	Newcastle University	S-1068836	D-1068836
DU145 (CVCL_0105)	Cellosaurus Database	N/A	N/A

Table of Allelic Data

	Genotypes		
STR Locus	DU145 (Test Sample)	DU145 (CVCL_0105) (Comparison Sample)	Match vs. Mis-Match
D5S818	10 | 13	10 | 13	Match
D13S317	12 | 14	12 | 13 | 14	Mis-Match
D7S820	7 | 10 | 11	7 | 10 | 11	Match
D16S539	11 | 12 | 13	11 | 13	Mis-Match
vWA	17 | 18	17 | 18 | 19	Mis-Match
TH01	7 | 7	7 | 7	Match
TPOX	11 | 11	11 | 11	Match
CSF1PO	10 | 11	10 | 11	Match
AMEL	X | Y	X | Y	Match
D3S1358	16 | 16	16 | 16	Match
D21S11	30 | 33	30 | 33	Match
D18S51	12 | 12	12 | 12	Match
Penta E	12 | 14	12 | 14	Match
Penta D	9 | 13	9 | 13	Match
D8S1179	13 | 14	13 | 14	Match
FGA	22 | 22	22 | 22	Match
D19S433	13 | 13	13 | 13	Match
D2S1338	16 | 17	16 | 16	Mis-Match

Matching Percentage: **94%**
Outcome: **Related**

The outcome percentage is calculated using the Tanabe algorithm. The outcome is designated one of the following statements based upon the outcome percentage:

Related (>80% match)	The Cell Lines are considered to be related.
Inconclusive (60-79% match)	Further profiling is required to determine whether the profiles are related.
No Match (59%>)	It is considered that the two cell lines are unrelated.

Reported By Ms. Ish Jones (Laboratory Scientist)

Authorised By Ms. Eleanor Ralston (Senior Scientist)

These test results should only be used in conjunction with a client's information. The results relate only to the items sampled, as received and tested at this time. DNA analysis as tested by PowerPlex® Fusion.

Cell Line Authentication Report for Case Number C-30186-4

We have now completed DNA analysis of the samples presented for Cell Line Authentication

A DNA Profile has been generated from the sample provided by Newcastle University and compared to the relevant profile located on the Cellosaurus Database. A Matching Percentage has been calculated and a value of 91% obtained. Please refer to the detailed results on the second page for further information and interpretation.

Analysis has been conducted using 17 loci allowing the probability of identity from identical matches an approximate power of discrimination of 1×10^{14} (1 in 100 trillion). Please note that some cell lines may exhibit genetic instability as they proliferate, leading to discrepancies within the DNA profiles examined.

Report Date 22/11/2023

This report shall not be reproduced, except in full, without written approval of the laboratory. The validity of these results depends on the quality of the sample provided and the identification of samples being correct.

northgene™
A BIOFORTUNA brand

UKAS TESTING 7816

Laboratory Report

Test Requested	Cell Line Authentication
Case Number	C-30186-4
Date Sample Received	13/11/2023
Date Sample Tested	13/11/2023
Date Sample Reported	22/11/2023

Sample Name / Cell Line	Cell Line Source / Profile Source	Sample Number	DNA Number
LNCAP	Newcastle University	S-1068837	D-1068837
LNCAP (CVCL_0395)	Cellosaurus Database	N/A	N/A

Table of Allelic Data

	Genotypes		
STR Locus	LNCAP (Test Sample)	LNCAP (CVCL_0395) (Comparison Sample)	Match vs. Mis-Match
D5S818	11 | 12	11 | 12	Match
D13S317	10 | 12	10 | 12	Match
D7S820	9.1 | 10.3	9.1 | 10.3	Match
D16S539	11 | 11	11 | 11	Match
vWA	16 | 18	16 | 18	Match
TH01	9 | 9	9 | 9	Match
TPOX	8 | 9	8 | 9	Match
CSF1PO	10 | 11	10 | 11	Match
AMEL	X | Y	X | Y	Match
D3S1358	16 | 16	16 | 16	Match
D21S11	29 | 31.2	29 | 31.2 | 32.2	Mis-Match
D18S51	11 | 12	11 | 12	Match
Penta E	12 | 15 | 16	12 | 16	Mis-Match
Penta D	12 | 12.4	12 | 12.4	Match
D8S1179	12 | 14	12 | 14	Match
FGA	19 | 21	19 | 20	Mis-Match
D19S433	13.2 | 15	14 | 15	Mis-Match
D2S1338	16 | 16	16 | 16	Match

Matching Percentage: **91%**
Outcome: **Related**

The outcome percentage is calculated using the Tanabe algorithm. The outcome is designated one of the following statements based upon the outcome percentage:

Related (>80% match)	The Cell Lines are considered to be related.
Inconclusive (60-79% match)	Further profiling is required to determine whether the profiles are related.
No Match (59%>)	It is considered that the two cell lines are unrelated.

Reported By Ms. Ish Jones (Laboratory Scientist)

Authorised By Ms. Eleanor Ralston (Senior Scientist)

These test results should only be used in conjunction with a client's information. The results relate only to the items sampled, as received and tested at this time. DNA analysis as tested by PowerPlex® Fusion.

Cell Line Authentication Report for Case Number C-30186-2

We have now completed DNA analysis of the samples presented for Cell Line Authentication

A DNA Profile has been generated from the sample provided by Newcastle University and compared to the relevant profile located on the Cellosaurus Database. A Matching Percentage has been calculated and a value of 100% obtained. Please refer to the detailed results on the second page for further information and interpretation.

Analysis has been conducted using 17 loci allowing the probability of identity from identical matches an approximate power of discrimination of $1x10^{14}$ (1 in 100 trillion). Please note that some cell lines may exhibit genetic instability as they proliferate, leading to discrepancies within the DNA profiles examined.

Report Date 22/11/2023

NorthGene™ is a trading name of Biofortuna Limited
2 Tenth Avenue Deeside Industrial Park Deeside CH5 2UA

This report shall not be reproduced, except in full, without written approval of the laboratory. The validity of these results depends on the quality of the sample provided and the identification of samples being correct.

northgene™
A BIOFORTUNA brand

UKAS TESTING 7816

Laboratory Report

Test Requested Cell Line Authentication

Case Number C-30186-2
Date Sample Received 13/11/2023
Date Sample Tested 13/11/2023
Date Sample Reported 22/11/2023

Sample Name / Cell Line	Cell Line Source / Profile Source	Sample Number	DNA Number
PC3	Newcastle University	S-1068835	D-1068835
PC-3 (CVCL_0035)	Cellosaurus Database	N/A	N/A

Table of Allelic Data

	Genotypes		
STR Locus	PC3 (Test Sample)	PC-3 (CVCL_0035) (Comparison Sample)	Match vs. Mis-Match
D5S818	13 | 13	13 | 13	Match
D13S317	11 | 11	11 | 11	Match
D7S820	FAIL	8 | 11	Mis-Match
D16S539	11 | 11	11 | 11	Match
vWA	17 | 17	17 | 17	Match
TH01	6 | 7	6 | 7	Match
TPOX	8 | 9	8 | 9	Match
CSF1PO	11 | 11	11 | 11	Match
AMEL	X | X	X | X	Match
D3S1358	16 | 16	16 | 16	Match
D21S11	29 | 31.2	29 | 31.2	Match
D18S51	14 | 15	14 | 15	Match
Penta E	10 | 17	10 | 17	Match
Penta D	9 | 9	9 | 9	Match
D8S1179	13 | 13	13 | 13	Match
FGA	24 | 24	24 | 24	Match
D19S433	14 | 14	14 | 14	Match
D2S1338	18 | 20	18 | 20	Match

Matching Percentage: **100%**
Outcome: **Related**

The outcome percentage is calculated using the Tanabe algorithm. The outcome is designated one of the following statements based upon the outcome percentage:

Related (>80% match) The Cell Lines are considered to be related.
Inconclusive (60-79% match) Further profiling is required to determine whether the profiles are related.
No Match (59%>) It is considered that the two cell lines are unrelated.

Reported By Ms. Ish Jones (Laboratory Scientist)

Authorised By Ms. Eleanor Ralston (Senior Scientist)

These test results should only be used in conjunction with a client's information. The results relate only to the items sampled, as received and tested at this time. DNA analysis as tested by PowerPlex® Fusion.
