# Supplementary figures and images for: Sialic acid blockade inhibits the metastatic spread of prostate cancer to bone
Source: eBioMedicine. 2024 May 20;104:105163. doi: 10.1016/j.ebiom.2024.105163 (PMC11134892; doi:10.1016/j.ebiom.2024.105163)

Fig. S2

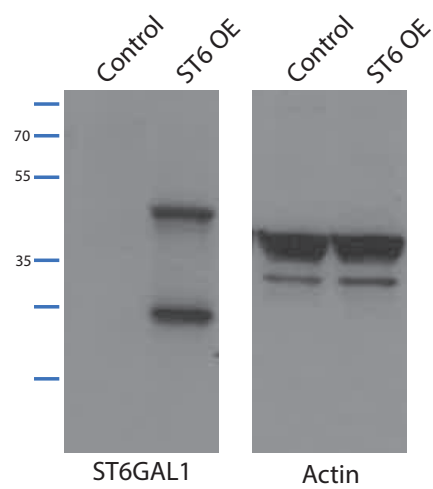

Fig. S6

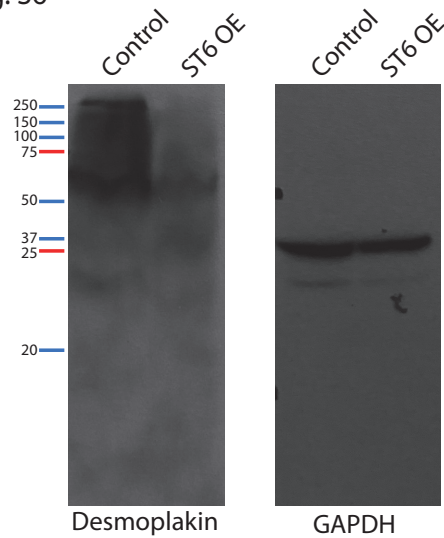

Supplement: Western blots [file mmc5.pdf]
